# The putative drug efflux systems of the *Bacillus cereus* group

**DOI:** 10.1371/journal.pone.0176188

**Published:** 2017-05-04

**Authors:** Karl A. Hassan, Annette Fagerlund, Liam D. H. Elbourne, Aniko Vörös, Jasmin K. Kroeger, Roger Simm, Nicolas J. Tourasse, Sarah Finke, Peter J. F. Henderson, Ole Andreas Økstad, Ian T. Paulsen, Anne-Brit Kolstø

**Affiliations:** 1 Department of Chemistry and Biomolecular Sciences, Macquarie University, Sydney, NSW, Australia; 2 School of BioMedical Sciences and Astbury Centre for Structural Molecular Biology, University of Leeds, Leeds, United Kingdom; 3 Laboratory for Microbial Dynamics (LaMDa), Section for Pharmaceutical Biosciences, School of Pharmacy, University of Oslo, Oslo, Norway; 4 Institut für Pharmazeutische Biologie und Biotechnologie, Albert-Ludwigs Universität, Freiburg, Germany; 5 Centre for Integrative Microbial Evolution (CIME), Faculty of Mathematics and Natural Sciences, University of Oslo, 0316 Oslo, Norway; University of Cambridge, UNITED KINGDOM

## Abstract

The *Bacillus cereus* group of bacteria includes seven closely related species, three of which, *B*. *anthracis*, *B*. *cereus* and *B*. *thuringiensis*, are pathogens of humans, animals and/or insects. Preliminary investigations into the transport capabilities of different bacterial lineages suggested that genes encoding putative efflux systems were unusually abundant in the *B*. *cereus* group compared to other bacteria. To explore the drug efflux potential of the *B*. *cereus* group all putative efflux systems were identified in the genomes of prototypical strains of *B*. *cereus*, *B*. *anthracis* and *B*. *thuringiensis* using our Transporter Automated Annotation Pipeline. More than 90 putative drug efflux systems were found within each of these strains, accounting for up to 2.7% of their protein coding potential. Comparative analyses demonstrated that the efflux systems are highly conserved between these species; 70–80% of the putative efflux pumps were shared between all three strains studied. Furthermore, 82% of the putative efflux system proteins encoded by the prototypical *B*. *cereus* strain ATCC 14579 (type strain) were found to be conserved in at least 80% of 169 *B*. *cereus* group strains that have high quality genome sequences available. However, only a handful of these efflux pumps have been functionally characterized. Deletion of individual efflux pump genes from *B*. *cereus* typically had little impact to drug resistance phenotypes or the general fitness of the strains, possibly because of the large numbers of alternative efflux systems that may have overlapping substrate specificities. Therefore, to gain insight into the possible transport functions of efflux systems in *B*. *cereus*, we undertook large-scale qRT-PCR analyses of efflux pump gene expression following drug shocks and other stress treatments. Clustering of gene expression changes identified several groups of similarly regulated systems that may have overlapping drug resistance functions. In this article we review current knowledge of the small molecule efflux pumps encoded by the *B*. *cereus* group and suggest the likely functions of numerous uncharacterised pumps.

## Introduction

The *Bacillus cereus* group is composed of seven species of low G+C Gram-positive spore-forming bacteria, which based on 16S rRNA sequence data form a separate cluster in the phylogenetic tree of *Bacillaceae* and Firmicutes [1]. The *B*. *cereus* group includes *B*. *cereus* (*sensu stricto*), *B*. *anthracis*, *and B*. *thuringiensis*, which are all well studied and are pathogens of animals, humans or insects, as well as *B*. *weihenstephanensis*, *B*. *mycoides*, *B*. *pseudomycoides* and *B*. *cytotoxicus*. The different species can commonly, but with variable frequency, be found in the soil environment, and can thus constitute polluter organisms in food production facilities and dairies, as well as in hospitals [2, 3]. Bacteria within the *B*. *cereus* group have also been suggested to naturally inhabit the insect gut [4].

The pathogenic species of the *B*. *cereus* group have different host preferences, mainly due to traits encoded on plasmids. *B*. *anthracis* is the cause of anthrax, primarily an animal disease but also occasionally of humans, due to its production of anthrax-specific toxins (lethal and edema toxins) and a poly-γ**-**D-glutamate capsule which provides protection against the host immune system. *B*. *anthracis* is endemic in several parts of the world [5]. The three toxin genes (*pag*, *lef* and *cya*) are located on a plasmid, pXO1 (189 kb), while the genes necessary for capsule synthesis, *capABCD*, are located on plasmid pXO2 (95 kb), and fully virulent *B*. *anthracis* strains carry both plasmids. *B*. *cereus sensu stricto* (here called *B*. *cereus*) is an opportunistic pathogen capable of causing a range of diseases [2, 6], most prominently foodborne disease due to the production of enterotoxins (diarrhoeal syndrome) or a non-ribosomally synthesized dodecadepsipeptide toxin (emetic syndrome). The emetic toxin is encoded by genes on a large 270 kb plasmid, pCER270 [7, 8]. Interestingly, *B*. *cereus* strains causing anthrax-like disease were isolated from welders in the US and shown to carry a plasmid highly similar to pXO1 [9], as well as from African great apes (Cameroon, Ivory Coast), shown to carry full pXO1 and pXO2 virulence plasmids [10, 11]. *B*. *thuringiensis* strains produce proteinaceous crystal toxins (Cry or Cyt toxin) during sporulation which are the primary cause of their toxicity toward insects, and which are encoded by genes most often located on plasmids. *B*. *thuringiensis* strains do however, also carry the chromosomal enterotoxin genes found in *B*. *cereus*, and the two species are genetically indistinguishable based on chromosomal characters [12, 13]. Many of the chromosomally encoded virulence factors in *B*. *cereus* and *B*. *thuringiensis* are positively regulated at the transcriptional level by the PlcR-PapR peptide-based quorum sensing system. The *plcR* gene is also present in *B*. *anthracis* strains, but carries a deleterious mutation making the protein non-functional and leaving the PlcR regulated genes non-transcribed [14].

Given that different species within the *B*. *cereus* group have diverse toxic effects and host specificities, but are closely related at the phylogenetic level, their intra- and inter-species diversity has frequently been studied at the genome level. Large-scale sequencing studies of *B*. *cereus* group strains have allowed the calculation of a core genome of genes shared between all strains (aproximately 1750 genes), and a set of additional genes found in almost every genome, constituting the extended core (approximately 2150 genes) [15]. The *B*. *cereus* group core genome appears to harbour a high number of genes encoding transporter proteins. This may reflect the fact that *B*. *cereus* group bacteria are frequently found in environments such as soil, which display high variability with respect to potential nutrients and exposure to toxic chemicals, including antibiotics and other antimicrobial agents. Putative efflux pumps appear to be particularly common within the genomes of the *B*. *cereus* group but relatively few of these transporters have been functionally characterised to date. In contrast, the model organism *Bacillus subtilis* encodes some of the best characterised multidrug efflux pumps in bacteria, including the related Bmr and Blt transporters from the major facilitator superfamily [16–18].

Bacterial drug efflux pumps generally fall into one of five families or superfamilies of transport proteins, the major facilitator superfamily (MFS), the ATP binding cassette (ABC) superfamily, the resistance/nodulation/division (RND) superfamily, the multidrug and toxic compound extrusion (MATE) family and the small multidrug resistance superfamily (SMR). A sixth family of multidrug efflux pumps, the Proteobacterial antimicrobial compound extrusion (PACE) family was recently identified [19, 20]. However, genes encoding PACE family proteins have been identified in the genome sequences of a small number of species outside the Proteobacteria.

Here we describe the putative efflux pumps carried by *B*. *cereus* group isolates that fall within each of the five major families of transport proteins. The number of pumps, their putative substrates and conservation across the group is described, followed by a detailed review of the efflux systems encoded by the *B*. *cereus* type strain, ATCC 14579. The transcriptional responses of selected conserved pumps encoded by this strain to a panel of structurally and mechanistically diverse drugs or stress conditions were determined to gain insight into their potential functional roles.

## Methods

### Bioinformatics analyses

Transport proteins encoded within the genomes of *B*. *cereus* ATCC 14579, *B*. *anthracis* Ames and *B*. *thuringiensis* konkukian 97–27, were identified using the Transporter Automated Annotation Pipeline (TransAAP) [21]. This pipeline predicts the complete complement of transporters encoded by an organism based on the annotated amino acid sequences within its genome sequence by running a variety of searches including BLASTP (to the Transporter Classification Database—TCDB, TransAAP and GenBank databases), HMM, Pfam, TIGRfam HMM and COG searches, as well as other analyses such as TMHMM hydropathy prediction [21]. Efflux proteins were identified in the TransAAP output and manually curated for a likely role in the efflux of drugs or small molecules.

To broadly examine the conservation of putative efflux systems between the *B*. *cereus* type strain ATCC 14579 and other strains within the *B*. *cereus* group, we conducted reciprocal best-match BLASTP 2.2.28+ analyses. Searches between all CDSs annotated in the ATCC 14579 genome and 168 other *B*. *cereus* group strains listed in the RefSeq database with assembly level “complete” or “chromosome” (August 2016; S1 Table) were executed through the Proteinortho tool [22]. Putative orthologs/paralogs were identified as reciprocal best-match BLASTP hits that recorded an e-value below 1e-50, and greater than 50% coverage. Since these analyses used annotated amino acid sequences as input, no pseudogenes were analysed.

### Antimicrobial exposure, stress treatments and RNA isolation

Minimum inhibitory concentrations (MIC) towards *B*. *cereus* ATCC 14579 for chloramphenicol, kanamycin, erythromycin, tetracycline, and ethidium bromide were previously determined [23], and MIC values for norfloxacin, 2,2’-dipyridyl, tannic acid, Dominulin B and a crude ethanol surface extract of a social paper wasp, *Polistes humilis* [24], were determined using the same method.

MH broth was inoculated with a 1% inoculum of an overnight culture of *B*. *cereus* ATCC 14579 and grown at 30°C with shaking to an OD_600_ of approximately 0.8. The culture was then diluted in MH broth to OD_600_ = 0.1, and grown as before to an OD_600_ of approximately 0.8. The culture was then split and the compound (or crude wasp ethanol extract) used for antimicrobial exposure treatment was added at a concentration equivalent to 50% of the respective MIC to separate cultures. An untreated culture was included as a control. The cultures were further grown for 20 minutes. Bacterial cells were harvested by incubating cultures in an equal volume of ice-cold methanol for 5 minutes before centrifugation at 4000 x *g* for 5 minutes. Pellets were stored at -80°C.

For extraction of RNA, cells were lysed using Lysing Matrix B and a FastPrep instrument (both MP Biomedicals), and RNA was isolated using the PureLink RNA Mini Kit (Invitrogen) or the RNeasy Mini Kit (Qiagen). RNA was treated with TURBO DNase (Ambion) as described, followed by a second round of purification using one of the RNA Mini Kits. RNA concentration and purity were measured using a NanoDrop ND-1000 spectrophotometer.

### Quantitative reverse transcription PCR (qRT-PCR)

cDNA synthesis was performed in duplicate for each RNA sample, using the SuperScript VILO cDNA Synthesis Kit (Invitrogen) or the Quantitect cDNA synthesis Kit (QIAGEN) and respective protocols, with 1μg RNA. qPCR reactions were performed on a MasterCycler realplex^4^ (Eppendorf) in a 96-well microtiterplate format and a final volume of 5μl using 1μl cDNA diluted 1:20, 2.5μl 2×GoTaq qPCR master mix (Promega) and 0.2μM of each primer. In qPCR experiments studying gene expression in cells exposed to wasp ethanol extract or Dominulin B, qPCR was performed in 200 ul thin-walled tubes and a final volume of 10μl, using 5.0μl 2×GoTaq qPCR master mix. Cycling conditions were 95°C for 2 minutes followed by 40 cycles at 95°C for 10 seconds, 55°C for 10 seconds, and 68°C for 8 seconds, followed by a melting curve analysis, which resulted in single product specific melting temperatures for all samples. Control qPCR reactions using DNase-treated RNA diluted to 0.005μg/μl as the template confirmed the absence of amplification of contaminating DNA.

Conserved putative MDR efflux pumps were selected for qPCR analysis after the stress treatments. After one round of qPCR only those conserved MFS and ABC genes that showed above 3-fold differential expression in at least one of the stress conditions were included in the further qPCR analysis (13 MFS and 6 ABC). In addition all the efflux pumps belonging to the RND, SMR and MATE families were included (three or four from each family).

The BC1744 helicase gene was selected for use as the reference gene. The list of primers used is given in S2 Table. For gene expression analysis, the quantification cycle (Cq) values were determined using the realplex software (Eppendorf). Cq values were transformed into linear scale expression quantities using the formula E^Cq^ [25]. The expression of each target gene was normalized to that obtained for the helicase reference gene reaction run on the same plate. Then, for each target gene, the expression ratio between the untreated and antimicrobial treated samples was calculated (ΔΔ-Cq-method) [25] and finally the values obtained for the two technical replicates were averaged.

### Biofilm formation

The biofilm forming capabilities of *B*. *cereus* ATCC 14579 wild type and isogenic markerless gene deletion mutant strains were investigated with a microplate screening assay modified from a previously described method [26]. Precultures were grown in Y1 minimal medium [27] at 3°C to early exponential growth (optical density at 600 nm (OD_600_) ~ 0.3) and were then used to inoculate fresh Y1 medium to an OD_600_ of 0.01. For each strain, sixteen wells of a 96-well polystyrene microplate (Corning 3788) were filled with 125 μl of the bacterial suspension. The plates were produced in duplicate and each plate contained eight wells of Y1 medium as a negative control. Following incubation at 20°C for 48 h and 72 h, respectively, the wells of each microplate were washed once with phosphate-buffered saline (PBS) and stained with a 0.1% (w/v) aqueous solution of methyl violet 6B for 30 min at room temperature. Wells were then washed three times with PBS and dried upside down over night. To quantify biofilm formation the dye was solubilized by incubating the wells with 150 μl of a 1:4 acetone/ethanol mixture for 10 min at room temperature, and subsequently absorbance at 570 nm was determined.

## Results and discussion

### Putative drug efflux systems are highly represented and well conserved in the *Bacillus cereus* group

To define the efflux potential of the *B*. *cereus* group, putative efflux systems were identified in the complete genome sequences of three reference strains, *B*. *cereus* ATCC 14579, *B*. *anthracis* Ames and *B*. *thuringiensis* konkukian 97–27, using the transporter automated annotation pipeline (TransAAP) [21]. These analyses identified 93, 93 and 103 putative efflux systems in these strains, respectively (Table 1). Remarkably, these efflux systems account for 2.3 to 2.7% of the predicted protein coding potential in these strains (Table 1). The majority of the efflux systems identified were classified within the MFS (greater than 50 pumps in all three strains) or ABC superfamily (28 to 35 transport systems), with only 3 to 5 efflux pumps from each of the RND, MATE and SMR (super)families (Table 1). For comparison, the numbers of putative efflux pumps encoded within the genomes of eight other representative bacterial strains within the Firmicutes were determined, including human and animal pathogens, and soil isolates that have similar lifestyles to the *B*. *cereus* group strains (Table 1). None of the other Firmicutes encoded as many putative efflux pumps as the *B*. *cereus* group isolates. However, the *Listeria* species examined, which have smaller genomes than strains in the *B*. *cereus* group, devoted a similar proportion of their protein coding potential to the production of transport proteins that may function in drug efflux as the *B*. *cereus* group strains (Table 1). These results suggest that strains in the *B*. *cereus* group have very high drug and/or small molecule efflux potential.

**Table 1:** Numbers of putative drug efflux systems encoded in the genomes of reference strains of the *B*. *cereus* group, and other Firmicutes.

Strain	ABC	MFS	MATE	SMR	RND	Total^*b*^	% ORFs
***Bacillus anthracis* Ames**	28^*a*^	51	4	5	4	**93**	**2.3**
***Bacillus cereus* ATCC 14579**	28	53	4	4	3	**93**	**2.3**
***Bacillus thuringensis* konkukian 97–27**	35	53	4	5	5	**103**	**2.7**
***Bacillus megaterium* QMB1551**	30	40	4	4	6	**84**	**1.7**
***Bacillus pumilus* SAFR032**	23	30	4	3	4	**64**	**2.0**
***Bacillus subtilis* 168**	20	39	4	4	4	**71**	**2.0**
***Staphylococcus aureus* N315**	16	22	1	1	2	**42**	**1.8**
***Clostridium perfringens* ATCC 13124**	16	8	10	0	1	**35**	**1.5**
***Listeria monocytogenes* La111**	27	23	9	1	3	**63**	**2.7**
***Listeria innocua* Clip11262**	31	18	6	1	2	**58**	**2.4**
***Geobacillus thermodenitrificans* NG802**	20	11	2	2	3	**38**	**1.5**

*a*. Transporters were identified using the Transporter Automated Annotation Pipeline and are listed at www.membranetransport.org.

*b*. Total number of transport systems. Some ABC and SMR (super)family systems are comprised of several proteins.

**Fig 1:**
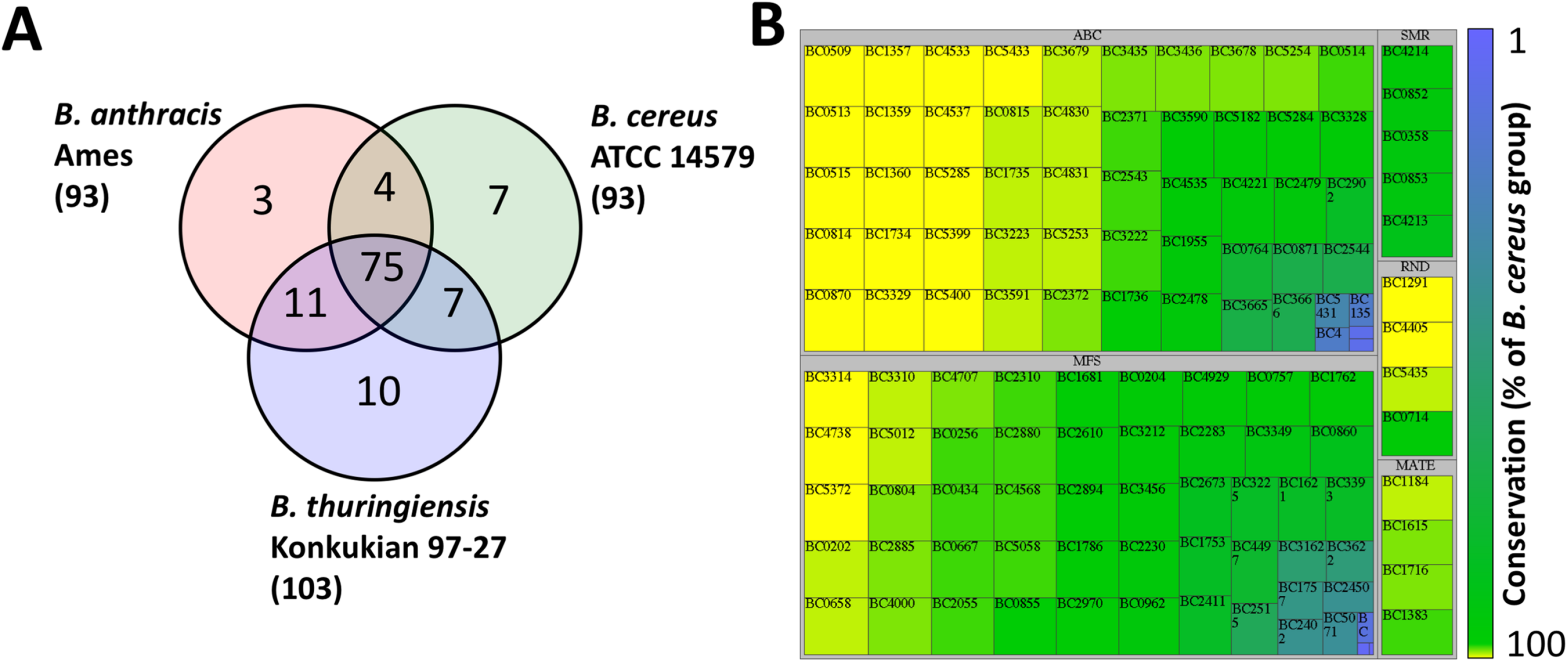
Conservation of putative efflux systems encoded in the *Bacillus cereus* group. (A) Venn diagram showing conservation of putative efflux systems in fully sequenced representatives of the *B*. *cereus* group. (B) Conservation of genes encoding efflux system components in *B*. *cereus* ATCC 14579. Reciprocal BLASTP 2.2.28+ searches (as executed through the Proteinortho tool [22]) of the *B*. *cereus* ATCC 14579 predicted proteome with 168 other strains in the *B*. *cereus* group (S1 Table) were used to determine the level of conservation. Each transporter component is represented by a single box, the size and shading of which corresponds to its conservation. Panel B was generated using TreeMap version 4.1.

To examine the level of conservation of the putative efflux systems in *B*. *cereus* ATCC 14579, *B*. *anthracis* Ames and *B*. *thuringiensis* konkukian 97–27, their predicted proteomes were compared using reciprocal best-match BLASTP searches. These searches suggested that 75 of the putative efflux systems were conserved in all three strains, representing 81% of those encoded in the *B*. *anthracis* Ames and *B*. *cereus* ATCC 14579 genomes (Fig 1A). To further explore the conservation of efflux systems in the *B*. *cereus* group, we examined the level of conservation of the *B*. *cereus* ATCC 14579 efflux pumps in 168 other *B*. *cereus* group strains with available high-quality genome sequences (S1 Table). This analysis suggested that 21 putative efflux proteins (components of 16 different efflux systems) encoded by *B*. *cereus* ATCC 14579 were conserved in all 168 strains (Fig 1B). Furthermore, 82% of the putative efflux system proteins in *B*. *cereus* ATCC 14579 were conserved in at least 80% of the strains examined (Fig 1B). These highly conserved putative efflux pumps are likely to have important core functions, possibly related to the basic physiology of the cell. The most poorly conserved transport systems were classified within the MFS or ABC superfamily (Fig 1B). However as mentioned above there are large numbers of these transporters encoded in *B*. *cereus* group genomes. Complete sets of putative transport proteins, including efflux pumps, encoded in the genomes of *B*. *cereus* group strains are listed in the TransportDB database [21].

### Major facilitator superfamily efflux pumps encoded in *B*. *cereus* ATCC 14579

The major facilitator superfamily (MFS) of transport proteins is an ancient protein family found in all classes of living organisms. MFS proteins participate in a broad range of transport reactions including the uptake of essential nutrients and the efflux of toxic compounds. Uptake and efflux pumps can be differentiated based on the presence of several key amino acid sequence motifs [28], such as sequence motif C which may be involved in the proton:substrate antiport coupling reaction [29]. The majority of bacterial drug efflux pumps classified within the MFS, are found within one of three transporter families, the drug:H^+^ antiport (DHA) 1–3 families, however, several other families are known or predicted to include drug efflux pumps. Proteins classified within the DHA1 and DHA3 families are typically organised into 12 transmembrane segments, similar to the majority of MFS pumps, whereas, those within the DHA2 family are typically organised into 14 transmembrane segments. DHA1 and DHA2 family protein sequences are more common in sequence databases and are encoded by both Gram-positive and Gram-negative bacteria, whereas, DHA3 family proteins are principally encoded by Gram-positives.

The genome of *B*. *cereus* ATCC 14579 encodes 53 putative MFS family drug efflux pumps. Thirty-eight of these transporters were predicted to fall within the DHA1, DHA2 or DHA3 families, 16, 12 and 10 proteins, respectively, based on BLASTP comparisons to all MFS proteins within the TCDB [30] (Table 2). The best hits for the remaining 15 putative *B*. *cereus* MFS efflux pumps were to three of the unknown major facilitator families (UMF2, UMF5 and UMF11), the nickel resistance (Nre) family, the putative aromatic compound/drug exporter (ACDE) family and the acriflavin-sensitivity (YnfM) family. Transporters within each of these families are known or predicted to function in the efflux of antimicrobial drugs.

**Table 2:** Putative *B*. *cereus* ATCC 14579 MFS efflux pumps.

Locus tag	Conservation^*a*^	Best match name	Function(s) of best match	Top blastp hit(s)^*b*^^,^^*c*^
**2.A.1.2—The Drug:H+ Antiporter-1 (12 Spanner) (DHA1) Family**				
BC0855*	97.6	Blt of *Bacillus subtilis*	Multidrug (and spermidine) efflux	P39843 **2.A.1.2.8** (0); P33449 **2.A.1.2.70** (6e-133); P0A0J7 **2.A.1.2.10** (3e-95)
**BC4738**	100.0	YttB of *Bacillus subtilis*	Unknown	O34546 **2.A.1.2.69** (4e-152); P0A0J7 **2.A.1.2.10** (4e-10); Q48658 **2.A.1.2.5** (3e-06)
**BC5012**	99.4	PbuE of *Bacillus subtilis*	Purine base/nucleoside efflux	Q797E3 **2.A.1.2.25** (8e-130); P77389 **2.A.1.2.65** (1e-40); Q9S3J9 **2.A.1.2.18** (5e-34)
BC1786*	97.0	MdtG of *Escherichia coli*	Putative multidrug efflux	P25744 **2.A.1.2.20** (1e-122); P0A4K4 **2.A.1.2.34** (7e-95); Q07282 **2.A.1.2.75** (8e-18)
**BC2402**	42.6	TetA42 of *Micrococcus* sp. SMCC G8878	Tetracycline resistance	B2YGG2 **2.A.1.2.41** (4e-72); P02982 **2.A.1.2.4** (9e-52); Q5JAK9 **2.A.1.2.39** (1e-49)
**BC3393**	82.8	YdhP of *Escherichia coli*	Unknown	P77389 **2.A.1.2.65** (1e-70); Q797E3 **2.A.1.2.25** (3e-57); P23910 **2.A.1.2.14** (7e-54)
**BC5058**	98.2	YdhP of *Escherichia coli*	Unknown	P77389 **2.A.1.2.65** (3e-70); Q797E3 **2.A.1.2.25** (2e-57); P23910 **2.A.1.2.14** (5e-55)
**BC3456**	95.3	EmrD-3 of *Vibrio cholerae*	Multidrug efflux	Q9KMQ3 **2.A.1.2.42** (1e-65); P32482 **2.A.1.2.3** (4e-26); Q7VW14 **2.A.1.2.27** (2e-24)
**BC0204**	96.4	Bcr of *Escherichia coli*	Multidrug (and L-cysteine) efflux	P28246 **2.A.1.2.7** (4e-65); Q7VW14 **2.A.1.2.27** (7e-39); P37597 **2.A.1.2.62** (5e-37)
**BC0860**	87.6	LmrP of *Lactococcus lactis*	Multidrug efflux	Q48658 **2.A.1.2.5** (8e-55); O34546 **2.A.1.2.69** (2e-15); P69367 **2.A.1.2.21** (3e-15)
BC0256*	98.2	YdeE of *Escherichia coli*	Peptide (and possibly arabinose) exporter	P31126 **2.A.1.2.55** (2e-20); B8GFY3 **2.A.1.46.4** (1e-20)
BC0667*	98.2	TetA41 of Serratia marcescens	Tetracycline exporter	Q5JAK9 **2.A.1.2.39** (2e-17); Q56RY7 **2.A.1.2.38** (2e-16); C2UR80 **2.A.1.46.5** (1e-14)
**BC3622**	51.5	YdeE of *Escherichia coli*	Peptide (and possibly arabinose) exporter	P31126 **2.A.1.2.55** (5e-22); O34546 **2.A.1.2.69** (7e-14); P69367 **2.A.1.2.21** (2e-13)
**BC2885**	98.8	TetA42 of *Micrococcus* sp. SMCC G8878	Tetracycline resistance	B2YGG2 **2.A.1.2.41** (5e-12); Q8NRB5 **2.A.1.2.24** (1e-12); P31126 **2.A.1.2.55** (3e-11)
**BC0202**	99.4	PmrA of *Streptococcus pneumoniae*	Multidrug efflux	P0A4K4 **2.A.1.2.34** (5e-09); P25744 **2.A.1.2.20** (1e-07); H6LDK2 **2.A.1.2.90** (1e-06)
**BC2061**	3.6	HsMDR of *Halobacterium* sp. NRC-1	Multidrug resistance	Q9HS33 **2.A.1.2.47** (5e-06)
**2.A.1.3—The Drug:H+ Antiporter-2 (14 Spanner) (DHA2) Family**				
BC4000*	98.8	Bmr3 of *Bacillus subtilis*	Multidrug resistance	P96712 **2.A.1.3.50** (0); O32182 **2.A.1.3.33** (1e-104); Q9ZGB6 **2.A.1.3.32** (9e-72)
**BC2880**	98.2	Bmr3 of *Bacillus subtilis*	Multidrug resistance	P96712 **2.A.1.3.50** (0); O32182 **2.A.1.3.33** (6e-101); Q9ZGB6 **2.A.1.3.32** (5e-66)
**BC0658**	99.4	MdtP of *Bacillus subtilis*	Multidrug efflux	O32182 **2.A.1.3.33** (0); P96712 **2.A.1.3.50** (6e-95); Q9ZGB6 **2.A.1.3.32** (1e-82)
**BC0962**	93.5	LmrB of *Bacillus subtilis*	Lincomycin resistance	O35018 **2.A.1.3.30** (2e-164); Q7A3S4 **2.A.1.3.61** (6e-109); Q5HE38 **2.A.1.3.39** (7e-99)
BC3212*	95.9	LmrB of *Bacillus subtilis*	Lincomycin resistance	O35018 **2.A.1.3.30** (6e-132); Q7A3S4 **2.A.1.3.61** (7e-117); Q5HE38 **2.A.1.3.39** (4e-111)
BC4568*	98.2	LmrB of *Bacillus subtilis*	Lincomycin resistance	O35018 **2.A.1.3.30** (2e-106); Q5HE38 **2.A.1.3.39** (4e-103); Q7A3S4 **2.A.1.3.61** (7e-93)
**BC0757**	95.9	YvmA of *Bacillus subtilis*	Unknown	O34307 **2.A.1.3.56** (3e-100); P37597 **2.A.1.2.62** (8e-26); O31762 **2.A.1.32.2** (3e-22)
BC4707*	98.8	Bmr3 of *Bacillus subtilis*	Multidrug resistance	P96712 **2.A.1.3.50** (2e-82); O32182 **2.A.1.3.33** (9e-80); Q9ZGB6 **2.A.1.3.32** (1e-64)
**BC1757**	46.2	EmrB of *Escherichia coli*	Multidrug efflux	P0AEJ0 **2.A.1.3.2** (9e-45); O32182 **2.A.1.3.33** (1e-44); Q9RQ29 **2.A.1.3.20** (1e-42)
**BC2310**	98.2	HsrA of *Escherichia coli*	Unknown	P31474 **2.A.1.3.51** (4e-53); O32182 **2.A.1.3.33** (2e-47); O35018 **2.A.1.3.30** (3e-44)
**BC4497**	79.3	TetA(L) of *Bacillus subtilis*	Me2+·tetracycline:2H+ antiporter	P23054 **2.A.1.3.16** (3e-46); P02983 **2.A.1.3.6** (7e-42); Q5PU79 **2.A.1.3.22** (8e-25)
**BC3349**	91.1	MdtH of *Escherichia coli*	Norfloxacin/enoxacin resistance	P69367 **2.A.1.2.21** (9e-30); O34546 **2.A.1.2.69** (7e-11); P0A0J7 **2.A.1.2.10** (2e-11)
**2.A.1.21—The Drug:H+ Antiporter-3 (12 Spanner) (DHA3) Family**				
**BC5071**	39.6	MefE of *Streptococcus pneumoniae*	Macrolide efflux	Q7BKK4 **2.A.1.21.22** (8e-52); P95827 **2.A.1.21.1** (1e-50); O31561 **2.A.1.31.3** (1e-19)
**BC2055**	98.2	YjbB of *Bacillus subtilis*	Unknown	O31600 **2.A.1.21.13** (5e-42)
**BC1621**	82.8	TIGR00900 of *Bacillus clausii*	Putative macrolide exporter	Q5WAS7 **2.A.1.21.8** (6e-32); O31561 **2.A.1.31.3** (2e-28); P39642 **2.A.1.21.5** (2e-18)
**BC1753**	84.0	TetV of *Mycobacterium smegmatis*	Tetracycline resistance	O31137 **2.A.1.21.3** (4e-25); C3WVU9 **2.A.1.62.2** (4e-17); Q0E7C5 **2.A.1.38.2** (1e-16)
**BC4929**	96.4	TetV of *Mycobacterium smegmatis*	Tetracycline resistance	O31137 **2.A.1.21.3** (1e-23); O31561 **2.A.1.31.3** (6e-17); A8YZ14 **2.A.1.62.1** (2e-17)
**BC2411**	83.4	MefE of *Streptococcus pneumoniae*	Macrolide efflux	Q7BKK4 **2.A.1.21.22** (2e-23); P95827 **2.A.1.21.1** (5e-21); C3WVU9 **2.A.1.62.2** (3e-20)
**BC2515**	63.9	MFS porter of *Stackebrandtia nassauensis*	Unknown	D3Q871 **2.A.1.21.11** (4e-21); O31561 **2.A.1.31.3** (1e-20); Q55937 **2.A.1.31.2** (1e-20)
BC0434*	98.2	TetV of *Mycobacterium smegmatis*	Tetracycline resistance	O31137 **2.A.1.21.3** (4e-19); O31561 **2.A.1.31.3** (2e-17); Q9X4X4 **2.A.1.30.1** (3e-13)
**BC3225**	83.4	MFS carrier of *Thermoplasma acidophilum*	Unknown	Q9HLP1 **2.A.1.21.9** (3e-17); Q9X4X4 **2.A.1.30.1** (4e-14); Q55937 **2.A.1.31.2** (3e-13)
**BC2325**	1.2	MefA of *Streptococcus pyogenes*	Macrolide efflux	P95827 **2.A.1.21.1** (1e-08); Q7BKK4 **2.A.1.21.22** (9e-07)
**2.A.1.26—The Unknown Major Facilitator-2 (UMF2) Family**				
BC3310*	99.4	YfkF of *Bacillus subtilis*	Possible drug exporter	O34929 **2.A.1.26.2** (2e-126); P21503 **2.A.1.26.1** (7e-16); Q56RY7 **2.A.1.2.38** (1e-09)
**2.A.1.31—The Nickel Resistance (Nre) Family**				
**BC2450**	42.0	KrsE of *Bacillus cereus*	Kurstakin/surfactin exporter ortholog	J8GQQ7 **2.A.1.31.4** (0); O31561 **2.A.1.31.3** (4e-38); O31137 **2.A.1.21.3** (7e-15)
BC1681*	97.6	YfiS of *Bacillus subtilis*	Unknown	O31561 **2.A.1.31.3** (1e-27); C3WVU9 **2.A.1.62.2** (1e-24); Q7BKK4 **2.A.1.21.22** (3e-21)
**BC2970**	97.0	NrsD of *Synechocystis* PCC6803	Ni2+ resistance protein	Q55937 **2.A.1.31.2** (2e-20); O31137 **2.A.1.21.3** (7e-16); Q7BKK4 **2.A.1.21.22** (2e-14)
BC2894*	97.6	YfiS of *Bacillus subtilis*	Unknown	O31561 **2.A.1.31.3** (2e-18); Q5WAS7 **2.A.1.21.8** (6e-13); P95827 **2.A.1.21.1** (1e-12)
**BC2610**	97.6	YfiS of *Bacillus subtilis*	Unknown	O31561 **2.A.1.31.3** (2e-17); Q5WGH2 **2.A.1.62.3** (7e-15); C3WVU9 **2.A.1.62.2 (**2e-12)
**2.A.1.32—The Putative Aromatic Compound/Drug Exporter (ACDE) Family**				
**BC5372**	100.0	YfmO of *Bacillus subtilis*	Putative copper/multidrug efflux	O06473 **2.A.1.32.3** (8e-83); Q54806 **2.A.1.3.5** (1e-18); P0A0J7 **2.A.1.2.10** (1e-18)
**2.A.1.35—The Fosmidomycin Resistance (Fsr) Family**				
**BC1762**	95.9	Fsr of *Escherichia coli*	Fosmidomycin, trimethoprim and CCCP	P52067 **2.A.1.35.1** (3e-97); Q56877 **2.A.1.35.2** (1e-78); F8IC89 **2.A.1.35.3** (5e-22)
**2.A.1.36—The Acriflavin-sensitivity (YnfM) Family**				
**BC3162**	54.4	YgaY of *Escherichia coli*	Unknown	P76628 **2.A.1.36.3** (1e-72); A8GHT9 **2.A.1.36.2** (2e-54); Q9ADP8 **2.A.1.36.4** (5e-34)
**2.A.1.46—The Unknown Major Facilitator-5 (UMF5) Family**				
**BC0804**	98.8	MFS porter of *Bacillus cereus*	Putative quinolone resistance	C2UR80 **2.A.1.46.5** (0); B8GFY3 **2.A.1.46.4** (2e-26); P0A0J7 **2.A.1.2.10** (3e-16)
**BC2283**	92.9	MFS porter of *Bacillus cereus*	Putative quinolone resistance	C2UR80 **2.A.1.46.5** (4e-104); B8GFY3 **2.A.1.46.4** (6e-24); P0A0J7 **2.A.1.2.10** (4e-19)
**BC3314**	100.0	MFS porter of *Bacillus cereus*	Putative quinolone resistance	C2UR80 **2.A.1.46.5** (2e-79); B8GFY3 **2.A.1.46.4** (1e-21); P37621 **2.A.1.46.7** (1e-18)
**2.A.1.62—The Unidentified Major Facilitator-11 (UMF11) Family**				
**BC2673**	85.8	P-MEP of *Fusobacterium* sp. 7_1	Putative Macrolide efflux, possibly amino acid transport	C3WVU9 **2.A.1.62.2** (2e-24); P95827 **2.A.1.21.1** (2e-23); Q7BKK4 **2.A.1.21.22** (2e-21)
BC2230*	94.1	UMF11 of *Staphylococcus aureus*	Unknown	A8YZ14 **2.A.1.62.1** (1e-18); P95827 **2.A.1.21.1** (9e-08); P64783 **2.A.1.21.12** (3e-07)
**BC3197**	12.4	P-MEP of *Fusobacterium* sp. 7_1	Putative Macrolide efflux, possibly amino acid transport	C3WVU9 **2.A.1.62.2** (7e-15); D3Q871 **2.A.1.21.11** (6e-11); Q55937 **2.A.1.31.2** (1e-11)

*a*. Numbers show the percent conservation of the protein in the predicted proteomes of 169 *B*. *cereus* group isolates according to comparative BLASTP searches (see Fig 1).

*b*. Uniprot accession numbers, TCDB accession numbers (boldface font) and e-values (in parentheses) for the top three blastp hits (e-value < 1e-5)

*c*. Blast hits for each family are in descending order of e-value for top hit

* genes marked with an asterisk were targeted by qRT-PCR analyses, see text for details.

Several MFS drug resistance efflux pumps have been previously characterised in *B*. *cereus*, including two members of the DHA2 family. The first of these, RZC03923 (orthologous to BC0962 in ATCC 14579) was cloned from *B*. *cereus* BRL1244, is similar to LmrB in *B*. *subtilis* and was characterised as part of a study examining the homologous DHA2 pump MdeA in *S*. *aureus* [31]. This pump was shown to confer resistance to virginiamycin, erythromycin, and lincomycin [31]. The second DHA2 family pump from *B*. *cereus* to be examined functionally, BC4707 from *B*. *cereus* ATCC 14579, was identified due to its increased expression in response to bile salts [32] and was found to facilitate resistance to norfloxacin, kanamycin and ciprofloxacin, and thus functions as a multidrug efflux pump [23]. In addition to the DHA2 family, a recent study by Kroeger *et al*. (2015) demonstrated that BC3310 encodes an active efflux pump that confers resistance to ethidium bromide, SDS and silver nitrate [33]. The BC3310 pump is the first protein from the UMF2 family of the MFS to have been studied experimentally, and its resistance phenotypes confirmed that members of the UMF2 family function in drug efflux [33].

Some *B*. *cereus* group MFS efflux pumps are likely to mediate the efflux of endogenously produced secondary metabolites. For example, BC2310 is located in a gene cluster coding for biosynthesis of bacillibactin [34], and is likely to mediate the efflux of this siderophore or a biosynthetic intermediate. BC2450 encodes an efflux pump that may transport a cyclic lipopeptide. Of the transporters listed in the TCDB, the BC2450 pump is most similar to the nickel resistance (Nre) family pump KrsE encoded by *B*. *cereus* VD014 (99% identical) (Table 2). The KrsE pump is encoded by the first gene in a large (~30 kb) six-gene cluster that includes several non-ribosomal peptide synthase genes involved in the biosynthesis of a cyclic lipopeptide, kurstakin. The cluster is also found in ATCC 14579 [35], but may not be active in this strain, possibly partly due to a transposon insertion in this strain in the quorum sensing regulator gene, *nprR*, which regulates production of kurstakin [36]. The role of KrsE in the efflux of kurstakin lipopeptides is yet to be demonstrated in *B*. *cereus* group strains, but a recent study demonstrated that an orthologous pump is involved in the efflux of a surfactin in *B*. *subtilis* [37]. Surfactin has been shown by a number of studies to be essential for formation of mature biofilms by *B*. *subtilis* [38, 39].

Several putative *B*. *cereus* MFS efflux pumps were very similar to characterised multidrug efflux pumps encoded by *B*. *subtilis* (e-value = 0; Table 2). These included the DHA1 family pump BC0855 (74% identity, 86% similarity to Blt), and the DHA2 family pumps BC4000 (62% identity, 76% similarity to Bmr3), BC2880 (60% identity, 76% similarity to Bmr3) and BC0658 (75% identity, 88% similarity to MdrP) (Table 2). Therefore, these *B*. *cereus* pumps may also mediate multidrug resistance.

Blt of *B*. *subtilis* was first recognised as being a multidrug efflux pump able to confer resistance to a range of substrates when overexpressed. Deletion of this gene from *B*. *subtilis* did not cause a decrease in antimicrobial resistance [40], possibly because *blt* has a low basal expression level and is not induced by antimicrobial substrates [16]. In addition to antimicrobials, the Blt multidrug efflux pump in *B*. *subtilis* is thought to have a physiological role in polyamine transport since the *blt* gene is encoded adjacent to a polyamine acetyltransferase gene and appears to promote the efflux of spermidine [41]. In contrast, the BC0855 gene is not encoded adjacent to a polyamine acetyltransferase gene, but is in a small cluster that also includes the SMR family transport protein genes BC0852 and BC0853 (see below), and a TetR family regulator gene BC0854. A partially palindromic sequence motif is conserved upstream of the BC0855 pump, the BC0854 regulator and the BC0852/BC0853 SMR pump genes with consensus: 5’-AAAaTGAxTGAtAGTCAtTCA-3’ (capital letters are in all three upstream regions, lower case in two and x is different in all). This may be a binding site for a regulatory protein, possibly that encoded by BC0854. Indeed, it was seen that in *B*. *anthracis* mutations in the orthologous regulator gene and/or its promoter region appeared to be responsible for derepression of all genes in the orthologous cluster. The increased expression of the transporter genes may have been responsible for ciprofloxacin resistance in *B*. *anthracis* [42]. A similar sequence (5’-AAAATAATTGACAGTCATTCA-3’) is found approximately 50 nt upstream of a putative biotin biosynthetic gene cluster (BC4120-BC4114) in the *B*. *cereus* ATCC 14579 genome, however, the relevance of this is unknown.

### ATP-binding cassette superfamily efflux pumps encoded in *B*. *cereus* ATCC 14579

Similar to the MFS the ABC superfamily of transport proteins is large and ancient, and ubiquitous to all classes of living organisms. In bacteria ABC superfamily pumps promote a range of both efflux and uptake transport reactions with substrates that include metabolites, vitamins, amino acids, lipids, peptides, ions and drugs. ABC superfamily pumps have been associated with drug resistance in bacteria and the cells of higher organisms, such as human cancer cells. The representative *B*. *cereus* group isolates examined in this work, *B*. *anthracis* Ames, *B*. *cereus* ATCC 14579 and *B*. *thuringensis* konkukian 97–27, each encoded between 28 and 35 ABC superfamily efflux pumps.

Comparisons of the ABC superfamily pumps identified in *B*. *cereus* ATCC 14579 with those in the TCDB using BLASTP identified several putative efflux systems that were closely related to previously characterised drug efflux pumps (e-value = 0; Table 3). These included two pumps that were similar to the YheI/YheH heterodimeric ABC superfamily multidrug efflux pump in *B*. *subtilis*, renamed as BmrC/BmrD [43, 44]; BC0870/BC0871 (65%/64% identity and 82%/80% similarity to the BmrC/BmrD), BC3679/BC3678 (48%/45% identity, 66%/67% similarity to BmrC/BmrD). In *B*. *subtilis* expression of BmrC/BmrD is responsive to ribosome-targeting antibiotics, and is controlled by a transcriptional attenuation mechanism that involves stem-loop structures upstream of *bmrC*, as well as a leader peptide BmrB which is encoded on the same transcript as *bmrC/bmrD* [45]. BC0870/BC0871 is most closely related to *bmrC/bmrD* in *B*. *cereus* ATCC 14579. BC0870 expression is also highly transcriptionally responsive to several ribosome targeting antibiotics (see below). The region upstream of BC0870 in *B*. *cereus* ATCC 14579 also contains sequences that could form stable stem-loop structures that may facilitate a similar mode of regulation in this strain. However, no clear homolog of BrmB is encoded in this region, highlighting a need for future experiments to investigate the regulation of BC0870/BC0871 in *B*. *cereus* group isolates.

**Table 3:** Putative *B*. *cereus* ATCC 14579 ABC efflux pumps.

Locus tag	Conservation^*a*^	Best match name	Function(s) of best match	Localisation^*b*^	Top blastp hit(s)^*c*^
**3.A.1.105: The Drug Exporter-1 (DrugE1) Family**					
**BC1734**	100.0	ABC2 of *Bacillus cereus*	Unknown	C	J8ABC0 **3.A.1.105.9** (2e-101); Q9A0K0 **3.A.1.105.7** (7e-93); Q7UE58 **3.A.1.105.8** (1e-67)
**BC1735**	99.4	SagGHI (Firmicutes)	May export streptolysin S	M	Q9A0J9 **3.A.1.105.7** (1e-37); J7ZHK9 **3.A.1.105.9** (1e-13); J8A8S6 **3.A.1.105.9** (1e-8)
**BC1736**	97.6	SagGHI (Firmicutes)	May export streptolysin S	M	Q9A0J8 **3.A.1.105.7** (1e-51); J7ZHK9 **3.A.1.105.9** (3e-34); J8A8S6 **3.A.1.105.9** (1e-15)
**BC2478**	94.1	ABC2 of *Bacillus cereus*	Unknown	C	J8ABC0 **3.A.1.105.9** (4e-63); Q3Z8A8 **3.A.1.105.6** (6e-62); Q4VWC9 **3.A.1.105.4** (3e-56)
**BC2479**	93.5	ABC-2 of *Dehalococcoides ethenogenes*	Unknown	M	Q3Z8A7 **3.A.1.105.6** (3e-54); P0AFP9 **3.A.1.105.15** (5e-15); Q4VWC7 **3.A.1.105.4** (6e-13)
**BC3435**	98.8	OleC5 of *Streptomyces antibioticus*	Drug resistance	M	Q53717 **3.A.1.105.2** (3e-31); P32011 **3.A.1.105.1** (2e-28); Q9F2Y7 **3.A.1.105.13** (3e-22)
**BC3436**	98.8	OleC4 of *Streptomyces antibioticus*	Drug resistance	C	Q53716 **3.A.1.105.2** (1e-75); Q9F2Y8 **3.A.1.105.13** (3e-74); P32010 **3.A.1.105.1** (6e-71)
**3.A.1.106: The Lipid Exporter (LipidE) Family**					
BC0509*	100.0	Sav1866 of *Staphylococcus aureus*	Multidrug resistance	MC	Q2G2M9 **3.A.1.106.2** (0); Q8G7R7 **3.A.1.106.3** (1e-120); Q9WYC4 **3.A.1.135.5** (4e-120)
BC0870*	100.0	YheI of *Bacillus subtilis*	Multidrug resistance	MC	O07550 **3.A.1.106.8** (0); P77265 3.**A.1.106.13** (1e-162); A7VN01 **3.A.1.106.**5 (2e-154)
**BC0871**	68.6	YheH of *Bacillus subtilis*	Multidrug resistance	MC	O07549 **3.A.1.106.8** (0); P0AAG5 **3.A.1.106.13** (1e-123); A7VN02 **3.A.1.106.5** (8e-113)
**BC3678**	98.8	YheH of *Bacillus subtilis*	Multidrug resistance	MC	O07549 **3.A.1.106.8** (9e-164); Q9WYC4 **3.A.1.135.5** (1e-142); A7VN02 **3.A.1.106.5** (5e-133)
**BC3679**	99.4	YheI of *Bacillus subtilis*	Multidrug resistance	MC	O07550 **3.A.1.106.8** (0); P77265 **3.A.1.106.13** (0); A7VN01 **3.A.1.106.5** (0)
BC5182*	97.0	Sav1866 of *Staphylococcus aureus*	Multidrug resistance	MC	Q2G2M9 **3.A.1.106.2** (8e-127); Q8G7R7 **3.A.1.106.3** (5e-112); Q9WYC4 **3.A.1.135.5** (4e-111)
**3.A.1.117: The Drug Exporter-2 (DrugE2) Family**					
**BC1955**	**94.7**	BmrA of *Bacillus subtilis*	Multidrug resistance	MC	O06967 **3.A.1.117.3** (0); P97046 **3.A.1.117.1** (5e-162); O32748 **3.A.1.117.2** (9e-162)
**3.A.1.122: The Macrolide Exporter (MacB) Family**					
**BC0764**	77.5	ABC transporter of *Methanocaldococcus jannaschii*	Unknown	C	Q58206 **3.A.1.122.14** (3e-67); O31711 **3.A.1.122.2** (1e-64); Q8RKC1 **3.A.1.122.3** (8e-64)
**BC0814**	100.0	YknZ of *Bacillus subtilis*	Antimicrobial peptide	M	O31712 **3.A.1.122.2** (2e-73); A0ZUB1 **3.A.1.122.12** (2e-48); P75831 **3.A.1.122.1** (6e-48)
**BC0815**	99.4	YknY of *Bacillus subtilis*	Antimicrobial peptide	C	O31711 **3.A.1.122.2** (5e-107); Q58206 **3.A.1.122.14** (8e-76); Q8RKC1 **3.A.1.122.3** (1e-73)
**BC3222**	98.2	HrtA of *Staphylococcus aureus*	Probable Heme exporter	C	Q7A3X3 **3.A.1.122.4** (8e-63); Q58206 **3.A.1.122.14** (6e-59); A8TDW7 **3.A.1.122.7** (9e-59)
**BC3223**	99.4	HrtB of *Corynebacterium diphtheriae*	Hemin resistance	M	H2GZC4 **3.A.1.122.11** (4e-28); Q8TM31 **3.A.1.122.6** (2e-7)
**BC5253**	99.4	YknZ of *Bacillus subtilis*	Antimicrobial peptide	M	O31712 **3.A.1.122.2** (3e-109); A0ZUB1 **3.A.1.122.12** (6e-59); P75831 **3.A.1.122.1** (9e-46)
**BC5254**	98.8	YknY of *Bacillus subtilis*	Antimicrobial peptide	C	O31711 **3.A.1.122.2** (4e-99); Q58206 **3.A.1.122.14** (7e-85); A8TDW7 **3.A.1.122.7** (2e-75)
**3.A.1.124: The 3-component Peptide-5 Exporter (Pep5E) Family**					
**BC4221**	94.1	SboF of *Streptococcus salivarius*	Salivaricin exporter	C	Q09II0 **3.A.1.124.5** (1e-40); Q75V15 **3.A.1.124.3** (5e-38); Q45404 **3.A.1.124.2** (1e-36)
**3.A.1.126: The β-Exotoxin I Exporter (βETE) Family**					
**BC3590**	97.6	BerB of *Bacillus thuringiensis*	Exporter of β-exotoxin I	M	Q8RME0 **3.A.1.126.1** (2e-175)
**BC3591**	99.4	BerA of *Bacillus thuringiensis*	Exporter of β-exotoxin I	C	Q8RME1 **3.A.1.126.1** (0); H8I779 **3.A.1.132.8** (3e-47); P42332 **3.A.1.131.1** (8e-47)
**3.A.1.132: The Gliding Motility ABC Transporter (Gld) Family**					
**BC2902**	83.4	ABC-2 of *Streptococcus pyogenes*	Unknown	C	Q99ZC8 **3.A.1.132.6** (1e-31); Q8RME1 **3.A.1.126.1** (1e-29); O30489 **3.A.1.132.1** (1e-28)
**3.A.1.134: The Peptide-7 Exporter (Pep7E) Family**					
**BC2543**	98.2	YxdL of *Bacillus subtilis*	Peptide/multidrug	C	P42423 **3.A.1.134.6** (8e-120); O06980 **3.A.1.134.5** (6e-115); Q8Y5F0 **3.A.1.134.12** (5e-97)
**BC2544**	68.0	YxdM of Bacillus subtilis	Peptide/multidrug	M	P42424 **3.A.1.134.6** (4e-116); O06981 **3.A.1.134.5** (9e-72); Q8Y5E9 **3.A.1.134.12** (7e-50)
**BC4823**	21.3	AnrB of *Listeria monocytogenes*	Multidrug resistance	M	Q8Y5E9 **3.A.1.134.12** (7e-141); Q8VUH1 **3.A.1.134.2** (2e-61); O34741 **3.A.1.134.3** (5e-61)
**BC4824**	0.0^*d*^	AnrA of *Listeria monocytogenes*	Multidrug resistance	C	Q8Y5F0 **3.A.1.134.12** (4e-65); O06980 **3.A.1.134.5** (2e-52); O34697 **3.A.1.134.3** (2e-50)
**BC4830**	99.4	AnrB of *Listeria monocytogenes*	Multidrug resistance	M	Q8Y5E9 **3.A.1.134.12** (2e-150); O06981 **3.A.1.134.5** (3e-64); O34741 **3.A.1.134.3** (8e-64)
**BC4831**	99.4	AnrA of *Listeria monocytogenes*	Multidrug resistance	C	Q8Y5F0 **3.A.1.134.12** (5e-125); O34697 **3.A.1.134.3** (1e-98); O06980 **3.A.1.134.5** (1e-95)
**3.A.1.135: The Drug Exporter-4 (DrugE4) Family**					
**BC2371**	98.2	TM287 of *Thermotoga maritima*	Unknown	MC	Q9WYC3 **3.A.1.135.5** (1e-175); B8ZPJ9 **3.A.1.135.4** (8e-137); G9CHY8 **3.A.1.135.3** (4e-136)
**BC2372**	98.8	TM288 of *Thermotoga maritima*	Unknown	MC	Q9WYC4 **3.A.1.135.5** (0); B8ZPD1 **3.A.1.135.4** (1e-145); Q8G7R7 **3.A.1.106.3** (3e-145)
**3.A.1.141: The Ethyl Viologen Exporter (EVE) Family (DUF990 Family)**					
**BC0513**	100.0	EvrA of *Synechocystis* sp. PCC6803	Ethyl viologen export	C	P73329 **3.A.1.141.1** (2e-85); Q8R6Q4 **3.A.1.141.2** (1e-65); P46903 **3.A.1.115.1** (5e-48)
**BC0514**	98.2	AbcB of *Thermoanaerobacter tengcongensis*	Unknown	M	Q8R6Q5 **3.A.1.141.2** (6e-21)
**BC0515**	100.0	EvrC of *Synechocystis* sp. PCC6803	Ethyl viologen export	M	P74757 **3.A.1.141.1** (2e-14); Q8R6Q6 **3.A.1.141.2** (9e-6);
**3.A.1.147:**					
**BC3328**	96.4	Exporter of *Natranaerobius thermophilus*	Unknown	M	B2A6N2 **3.A.1.147.5** (2e-9); J7IPE5 **3.A.1.147.10** (4e-9); C9XJW9 **3.A.1.147.6** (9e-8)
**BC3329**	100.0	Exporter of *Clostridium difficile*	Unknown	C	C9XJX0 **3.A.1.147.6** (3e-88); C1A6K8 **3.A.1.147.1** (3e-75); B8ZKM9 **3.A.1.147.8** (1e-74)
**No clear family**					
**BC1357**	100.0	ABC-2 of *Streptococcus pyogenes*	Unknown	C	Q99ZC8 **3.A.1.132.6** (7e-68); P46903 **3.A.1.115.1** (5e-30); Q2SDB1 **3.A.1.132.4** (5e-29)
**BC1358**	20.7	NA	NA		**no significant hits**
BC1359*	100.0	SboF of *Streptococcus salivarius*	Salivaricin exporter	C	Q09II0 **3.A.1.124.5** (4e-65); P42332 **3.A.1.131.1** (1e-62); Q75V15 **3.A.1.124.3** (2e-60)
**BC1360**	100.0	NA	NA		**no significant hits**
**BC2719**	7.7	SboF of *Streptococcus salivarius*	Salivaricin exporter	C	Q09II0 **3.A.1.124.5** (3e-56); Q75V15 **3.A.1.124.3** (3e-55); P42332 **3.A.1.131.1** (1e-50)
**BC2720**	7.7				**no significant hits**
**BC3665**	70.4	NA	NA		**no significant hits**
**BC3666**	66.9	SboF of *Streptococcus salivarius*	Salivaricin exporter	C	Q09II0 **3.A.1.124.5** (1e-69); A6MER5 **3.A.1.124.4** (2e-64); Q75V15 **3.A.1.124.3** (2e-62)
**BC4533**	100.0	NA	NA		**no significant hits**
**BC4535**	96.4	NA	NA		**no significant hits**
**BC4537**	100.0	BcrA of *Bacillus licheniformis*	bacitracin resistance	C	P42332 **3.A.1.131.1** (3e-94); Q09II0 **3.A.1.124.5** (1e-68); Q75V15 **3.A.1.124.3** (2e-65)
**BC5284**	97.0	PltJ of P*seudomonas* sp. M18	Polyketide efflux	M	Q4VWC8 **3.A.1.105.4** (3e-6)
BC5285*	100.0	ABC2 #2 of *Methanocella arvoryzae*	Unknown	C	Q0W8T7 **3.A.1.144.2** (8e-56); J8ABC0 **3.A.1.105.9** (7e-53); Q0W8T4 **3.A.1.144.1** (3e-52)
**BC5399**	100.0	NatB of *Rhodopirellula baltica*	Na extrusion (putative)	M	Q7UQ82 **3.A.1.115.2** (1e-7); Q7NL24 **3.A.1.132.10** (5e-6);
**BC5400**	100.0	BcrA of *Bacillus licheniformis*	Bacitracin resistance	C	P42332 **3.A.1.131.1** (7e-80); Q09II0 **3.A.1.124.5** (2e-69); H8I779 **3.A.1.132.8** (4e-67)
**BC5431**	31.4	NA	NA		**no significant hits**
BC5433*	100.0	CmpA of *Clostridium hathewayi*	Drug transport	M	Q83XH1 **3.A.1.121.4** (1e-54); P43672 **3.A.1.120.6** (3e-54); Q60248 **3.A.1.120.4** (2e-47)

*a*. Numbers show the percent conservation of the protein in the predicted proteomes of 169 *B*. *cereus* group isolates according to comparative BLASTP searches (see Fig 1).

*b*. Localization, M: transmembrane domain, C: cytoplasmic ATP-binding domain, MC: fused membrane and cytoplasmic domains.

*c*. Uniprot accession numbers, TCDB accession numbers (boldface font) and e-values (in parentheses) for the top three blastp hits (e-value < 1e-5).

*d*. BC4824 is annotated as a pseudogene, and is thus not associated with a protein coding sequence.

* genes marked with an asterisk were targeted by qRT-PCR analyses, see text for details.

Three other ABC efflux systems identified in *B*. *cereus* ATCC 14579 were also closely related to previously characterised drug efflux pumps listed in the TCDB and may function in drug efflux. These include, BC1955 (63% identity, 78% similarity to BmrA of *Bacillus subtilis*), BC0509 (59% identity, 78% similarity to Sav1866 of *Staphylococcus aureus*), and BC2371/BC2372 (45%/46% identity, 66%/66% similarity to TM287/TM288 of *Thermotoga maritima*).

The transporter encoded by BC3590/BC3591 is orthologous to the BerA/BerB transport system of *B*. *thuringensis* (95%/99% Identity, 97%/99% similarity), which has been linked to β-exotoxin production/efflux [46]. The organisation of genes adjacent to BC3590/BC3591 is identical in *B*. *cereus* ATCC 14579 and the β-exotoxin producing strain *B*. *thuringiensis* 407–1 [47]. Therefore, the regulation of BC3590/BC3591 in *B*. *cereus* ATCC 14579 may be similar to *berA/berB* in *B*. *thuringiensis*. However, *B*. *cereus* ATCC 14579 does not produce β-exotoxin, so the function of the pump encoded by BC3590/BC3591 is unknown. Genes encoding BerA/BerB orthologs are conserved in 97.6–99.4% of *B*. *cereus* group isolates (Fig 1B; Table 3), therefore this ABC pump may have a core physiological function, potentially playing a fortuitous role in β-exotoxin transport in strains that produce this toxin.

### Resistance/nodulation/division superfamily efflux pumps encoded in *B*. *cereus* ATCC 14579

Transport proteins classified within the RND superfamily of efflux pumps facilitate the efflux of diverse substrates including antimicrobials, metals and lipids. Specialised RND pumps within the SecDF family form accessory components of the Sec-translocase and thus participate in protein secretion. In Gram-negative bacteria most RND pumps that mediate small molecule transport are thought to form complexes with membrane fusion proteins and outer-membrane proteins that allow substrates to be captured within the periplasm or outer leaflet of the inner-membrane and transported across the outer-membrane. For example, the periplasmic head domain in the AcrB RND pump from *E*. *coli* docks with the TolC outer-membrane protein and the AcrA membrane fusion protein to move substrates across the outer-membrane [48]. It remains to be demonstrated whether RND pumps are able to capture substrates from within the bacterial cytoplasm. Since Gram-positive bacteria do not have an outer-membrane, the substrates and molecular transport mechanisms of Gram-positive RND efflux pumps, such as those encoded by strains within the *B*. *cereus* group, are of particular interest.

The genome of *B*. *cereus* ATCC 14579 encodes four RND superfamily transporters, BC0714, BC1291, BC4405 and BC5435. One of these proteins, BC4405, has been studied previously by members of our team and shown to encode the SecDF component of the Sec-translocase [49]. BLASTP and phylogenetic analyses conducted here confirmed the relationship of BC4405 and other SecDF RND proteins within the SecDF family (TCDB 2.A.6.4) (Table 4). The functions of the remaining three RND proteins in *B*. *cereus* ATCC 14579 are unknown, but may involve drug efflux (Table 1). Each of these proteins is highly conserved in at least 96% of sequenced representatives in the *B*. *cereus* group (Table 4; Fig 1), suggesting an important core function (Table 4).

**Table 4:** Putative *B*. *cereus* ATCC 14579 RND efflux pumps.

Locus tag	Conservation^*a*^	Best match name	Function(s) of best match	Top blastp hit(s)^*b*^
**BC_0714**	96.4	YerP of *Bacillus subtilis*	Surfactin export	D4G632 **2.A.6.3.9** (0); Q8CX78 **2.A.6.3.6** (6e-128); B4WH09 **2.A.6.3.5** (4e-116)
**BC_1291**	100.0	MmpL3 of *Mycobacterium tuberculosis*	Trehalose monomycolate export	O53657 **2.A.6.5.6** (2e-77); P65374 **2.A.6.5.5** (3e-35); Q53902 **2.A.6.5.1** (3e-34)
**BC_4405**	100.0	SecDF of *Bacillus subtilis*	Protein translocation	O32047 **2.A.6.4.2** (0); Q5SKE6 **2.A.6.4.3** (3e-102); P0AG90 **2.A.6.4.1** (2e-43)
**BC_5435**	99.4	YerP of *Bacillus subtilis*	Surfactin export	D4G632 **2.A.6.3.9** (0); Q8CX78 **2.A.6.3.6** (7e-149); Q1DEX6 **2.A.6.3.4** (2e-135)

*a*. Numbers show the percent conservation of the protein in the predicted proteomes of 169 *B*. *cereus* group isolates according to comparative BLASTP searches (see Fig 1).

*b*. Uniprot accession numbers, TCDB accession numbers (boldface font) and e-values (in parentheses) for the top three blastp hits (e-value < 1e-5).

BLASTP and phylogenetic analyses showed that the BC0714 and BC5435 pumps should be classified as members of the putative nodulation factor exporter (NFE) family (TCDB 2.A.6.3) and are most closely related to YerP from *B*. *subtilis* (Table 4). Functional analyses of YerP recently demonstrated that overexpression of this pump in its native host resulted in increased secretion of endogenously produced surfactin into the supernatant [37]. YerP is also known to be involved in surfactin resistance in strains that do not produce an endogenous surfactin and can mediate resistance to acriflavine and ethidium [50]. Amphiphilic substrates such as surfactin, acriflavine or ethidium could be present in the outer-leaflet of the cytoplasmic membrane in *Bacillus* species and be stripped from this location by an RND pump, then expelled into the environment. Similar to YerP, BC0714 and BC5435 may recognise an endogenous substrate. A noteworthy feature of the BC5435 sequence was the presence of an extended periplasmic loop in the region corresponding to the TolC docking domain of the structurally characterised AcrB pump (S1 Fig). An extended loop is also present in the *B*. *subtilis* YerP protein, but not in any of the other RND proteins currently listed in TCDB. The loop in BC5435 is glutamine, serine and alanine-rich which may be important for function, possibly playing a role in substrate release given the putative location of the loops near the substrate exit site.

The fourth RND pump encoded by *B*. *cereus* ATCC 14579, BC1291, fell within the (Gram-positive bacterial putative) hydrophobe/amphiphile efflux-2 (HAE2) family (TCDB 2.A.6.5) clade (Table 4). Most of the characterised pumps in this family transport lipids or cell wall components. With respect to proteins listed in the TCDB, BC1291 is most related to MmpL3 and MmpL11 from *Mycobacterium tuberculosis*, which transport mycobacterial specific cell wall components (Table 4). The YdfJ system encoded in *B*. *subtilis* is also a member of the HAE2 family. A deletion mutant of this pump did not show increased susceptibility to a panel of more than 31 antimicrobials [51]. Therefore, these pumps may not have any cross-specificity for drugs.

### Small multidrug resistance family efflux pumps encoded in *B*. *cereus* ATCC 14579

The SMR family is classified within the drug/metabolite superfamily, which also includes families of pumps that mediate the export or uptake of a range of sugars, amino acids and other metabolites. Transporters classified within the SMR family are the smallest known efflux pumps that have been characterised to date. A complete SMR transport system consists of two polypeptides, each approximately 110 amino acids in length, and can be homo- or heterodimeric. There are three putative SMR family transport systems encoded in the genome of *B*. *cereus* ATCC 14579. Two of these pumps, BC0852/BC0853 and BC4213/BC4214, are predicted to function as heterodimers, since they are each encoded by two adjacent genes. These two systems are homologous to the *B*. *subtilis* YkkCD system (Table 5). The complete YkkCD transporter is a multidrug efflux pump that confers resistance to a range of antibiotics and biocides [52]. As mentioned above BC0852/BC0853 are encoded near the *blt* homolog BC0855 in the *B*. *cereus* genome and are likely to be under similar regulatory control to this pump. The third SMR efflux pump encoded by *B*. *cereus* ATCC 14579, BC0358, is likely to function as a homologomer and is most related to NepA of *Arthrobacter nicotinovorans* (37% identity, 55% similarity), part of the NepAB efflux pump, and the staphylococcal QacC pump (35% identity, 63% similarity). The NepAB system is predicted to export methylamine [53], whereas QacC is a prototypical member of the SMR family and confers resistance to a range of cationic biocides [54].

**Table 5:** Putative *B*. *cereus* ATCC 14579 SMR efflux pumps.

Locus tag	Conservation^*a*^	Best match name	Function(s) of best match	Top blastp hit(s)^*b*^
**BC0358**	92.9	NepA of *Arthrobacter nicotinovorans*	probably exports methylamine	Q8GAI5 **2.A.7.1.8** (2e-20); P14319 **2.A.7.1.1** (6e-20); Q2FD83 **2.A.7.1.11** (1e-18)
**BC0852**	93.5	YkkC of *Bacillus subtilis*	Multidrug efflux	P49856 **2.A.7.1.5** (1e-14); D5CES3 **2.A.7.1.10** (2e-13); P69937 **2.A.7.1.4** (3e-12)
**BC0853**	92.3	YkkD of *Bacillus subtilis*	Multidrug efflux	P49857 **2.A.7.1.5** (7e-21); D5CES3 **2.A.7.1.10** (3e-20); P69937 **2.A.7.1.4** (1e-17)
**BC4213**	88.2	YkkC of *Bacillus subtilis*	Multidrug efflux	P49856 **2.A.7.1.5** (4e-27); D5CES3 **2.A.7.1.10** (1e-22); P69937 **2.A.7.1.4** (3e-21
**BC4214**	95.3	YkkD of *Bacillus subtilis*	Multidrug efflux	P49857 **2.A.7.1.5** (4e-32); D5CES3 **2.A.7.1.10** (4e-27); P69937 **2.A.7.1.4** (6e-25)

*a*. Numbers show the percent conservation of the protein in the predicted proteomes of 169 *B*. *cereus* group isolates according to comparative BLASTP searches (see Fig 1).

*b*. Uniprot accession numbers, TCDB accession numbers (boldface font) and e-values (in parentheses) for the top three blastp hits (e-value < 1e-5).

### Multidrug and toxic compound extrusion family efflux pumps encoded in *B*. *cereus* ATCC 14579

The MATE family of multidrug efflux pumps is one of 31 families classified within the multidrug/oligosaccharidyl-lipid/polysaccharide flippase superfamily. Transport proteins classified within the MATE family are ubiquitous to all classes of living organisms and are energised by secondary energy sources, including the proton- or sodium-motive-force. The genome of *B*. *cereus* ATCC 14579 encodes four putative MATE family efflux pumps, BC1184, BC1383, BC1615 and BC1716, each of which is conserved in more than 98% of the *B*. *cereus* group strains to have had their genome sequences determined (Fig 1). None of the *B*. *cereus* ATCC 14579 MATE pumps have been functionally characterised. The pump encoded by BC1716 is very similar (75% identity, 89% similarity) to the putative multidrug efflux system, YoeA from *B*. *subtilis* (Table 6). The pump encoded by BC1615 is related to DinF from *Bacillus halodurans* (31% identity, 56% similarity). DinF is multidrug efflux pump that was recently characterised by X-ray crystallography, providing details of the substrate binding site and proton coupling mechanism [55]. The BC1615 pump may also act as a multidrug efflux pump and recognise similar substrates to DinF, including the antimicrobial dyes ethidium and rhodamine 6G [55].

**Table 6:** Putative *B*. *cereus* ATCC 14579 MATE efflux pumps.

Locus tag	Conservation^*a*^	Best match name	Function(s) of best match	Top blastp hit(s)^*b*^
**BC1184**	99.4	NorM of *Thermotoga maritima*	Probable multidrug resistance	Q9WZS2 **2.A.66.1.28** (8e-44); P76352 **2.A.66.1.23** (2e-37); D5CJ69 **2.A.66.1.22** (5e-32)
**BC1383**	98.2	PdrM of *Streptococcus pneumoniae*	Multidrug efflux	Q8DPQ6 **2.A.66.1.41** (1e-100); Q9I3Y3 2**.A.66.1.12** (2e-97); O82855 **2.A.66.1.1** (5e-95)
**BC1615**	98.8	DinF-like pump of *Bacillus halodurans*	Multidrug efflux	Q9KAX3 **2.A.66.1.32** (1e-67); Q7WZ38 **2.A.66.1.37** (2e-64); Q93HR7 **2.A.66.1.7** (3e-50)
**BC1716**	98.8	YoeA of *Bacillus subtilis*	Probable multidrug resistance	O34474 **2.A.66.1.25** (0); Q2G140 **2.A.66.1.13** (3e-33); I6L8P4 **2.A.66.1.33** (4e-33)

*a*. Numbers show the percent conservation of the protein in the predicted proteomes of 169 *B*. *cereus* group isolates according to comparative BLASTP searches (see Fig 1).

*b*. Uniprot accession numbers, TCDB accession numbers (boldface font) and e-values (in parentheses) for the top three blastp hits (e-value < 1e-5).

### Large scale qRT-PCR analyses to examine potential physiological functions of efflux pumps in *B*. *cereus* ATCC 14579

To experimentally characterise the efflux functions of pumps identified in our *in silico* analyses, we have constructed a number of gene deletion mutants. To date we have made targeted deletions in three genes encoding MFS pumps, BC4707 [23], BC3310 [33] and BC4000, all four genes encoding RND pumps, BC 0714, BC1291, BC4405 [49] and BC5435, as well as BC1360 and BC0852, which encode components of an ABC pump and an SMR pump, respectively. The construction of *B*. *cereus* gene deletion mutants is labour intensive and this work identified drug resistance phenotypes for only two of the targeted pumps [23, 33], possibly because of functional redundancy between sub-sets of pumps encoded in *B*. *cereus*, due to overlapping substrate specificities. Furthermore, a loss-of-function screen for reduced biofilm formation among deletion mutants in transporters included in this study identified BC4405 as the only transporter with an identifiable phenotype (S2 Fig), in line with the role of SecDF in protein secretion, and the importance of cell surface proteins in *B*. *cereus* group biofilm formation [56]. To assess the potential transport functions of putative efflux systems in *B*. *cereus* with increased throughput, we adopted an alternative approach based on gene expression.

Most efflux pumps are only required by bacterial cells at specific times, e.g., when their substrates reach a threshold level in the cell, and the uncontrolled expression of efflux pumps at other times could reduce cellular fitness. Consequently, efflux pump expression can be tightly controlled in response to substrate or substrate-related environmental stress conditions. This inducible regulatory control offers a potential mechanism to gain insight into the core physiological functions of efflux pumps by evaluating transcriptional responses to putative substrates by qRT-PCR. To this end, we evaluated the expression of 30 efflux system genes in *B*. *cereus* ATCC 14579 after exposure to a panel of nine antimicrobials or stress conditions. The efflux systems tested included all three SMR family (Table 5), all four MATE family (Table 6) and all four RND superfamily (Table 4) pumps identified in this strain, as well as, 13 MFS (Table 2) and six ABC superfamily pumps (Table 3).

Of the eight compounds tested, five were antibiotics belonging to different drug classes that are likely to be transported by efflux pumps, i.e., chloramphenicol, norfloxacin, kanamycin, erythromycin and tetracycline. The antimicrobial dye ethidium bromide was included as it is a common substrate for multidrug efflux pumps. The iron-chelating compound 2,2’-dipyridyl (DIP) was included to promote iron limitation and highlight efflux systems that may be involved in iron homeostasis. Tannic acid, a polyphenolic plant derived compound was included as an environmental compound with antimicrobial properties. Finally, an extract from the cuticle of the common paper wasp *Polistes humilis*, shown to have antimicrobial activity [24], was included. This wasp extract is likely to contain a mixture of antimicrobial compounds produced by the insect to provide microbial defence. The susceptibility of *B*. *cereus* ATCC 14579 towards the compounds was determined (S3 Table), and the cells treated with concentrations 50% of their respective minimum inhibitory concentrations (MIC).

**Fig 2:**
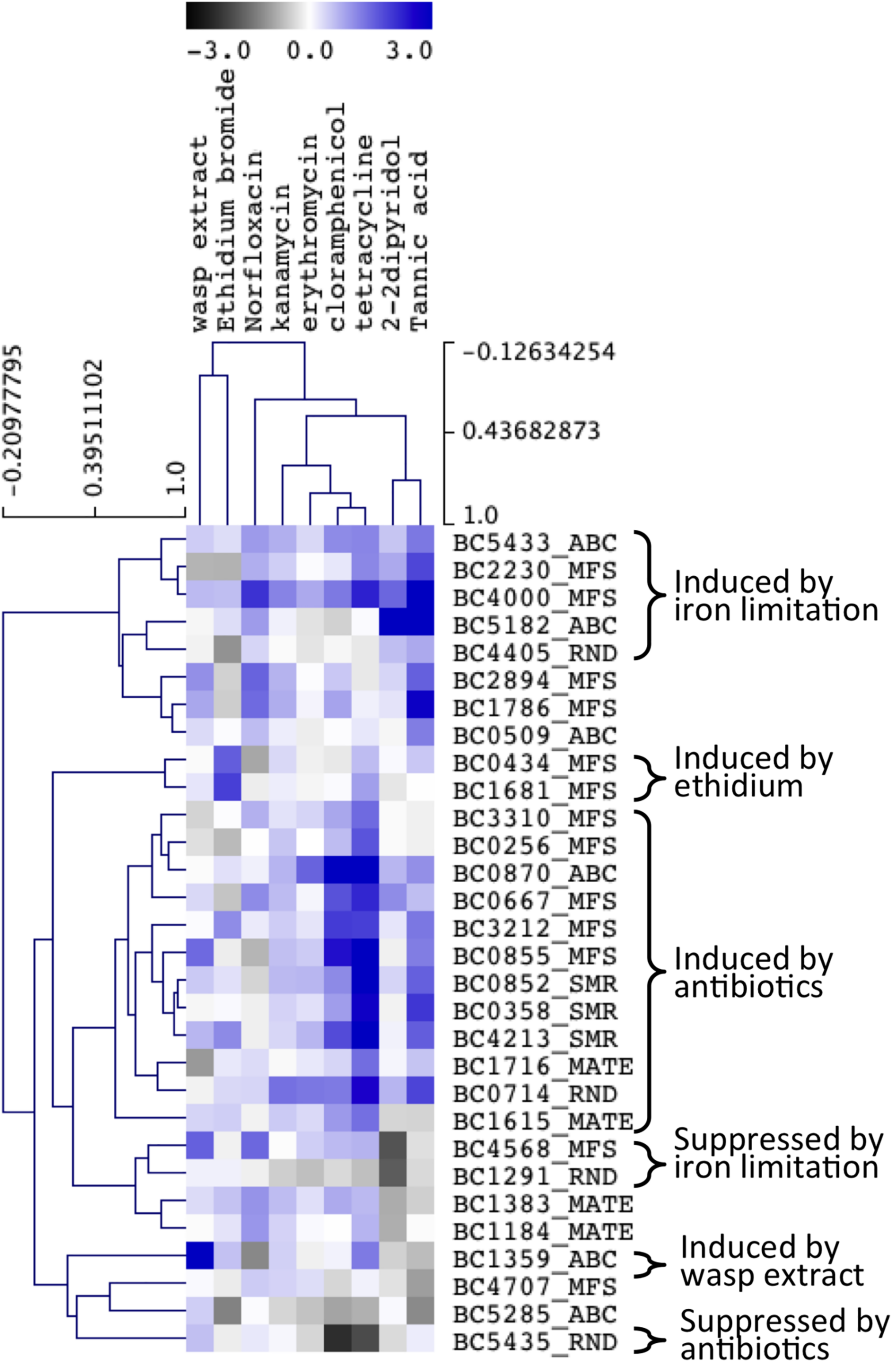
Gene expression changes in response to antimicrobial and environmental shock treatments. Relative efflux pump gene expression levels were examined using qRT-PCR on RNA extracted from *B*. *cereus* ATCC 14579 treated with antimicrobial compounds compared with untreated cells. *B*. *cereus* ATCC 14579 cells were grown at 30°C in MH broth to OD_600_ = 0.8 and then treated for 20 minutes with the antimicrobial compounds chloramphenicol, norfloxacin, kanamycin, erythromycin, tetracycline, ethidium bromide, 2,2’-dipyridole, tannic acid and wasp extract, at concentrations corresponding to 50% of the MIC (S3 Table). The BC1744 helicase gene was used as the reference gene to normalize the data. Hierarchical clustering analysis [57] was performed on the average gene expression values using the Pearson correlation method using the TIGR Multi-Experiment Viewer TMEV software [58]. The scale shows log2 fold-changes in gene expression between treated cells and untreated controls.

We conducted hierarchical clustering to identify compounds that induced similar expression responses among the genes, and conversely sub-sets of genes that showed similar patterns of expression in response to the different antimicrobials (Fig 2). These analyses indicated that the antibiotics, particularly kanamycin, erythromycin, chloramphenicol and tetracycline, induced similar changes in gene expression. Tannic acid and DIP also induced a similar pattern of induction across the genes tested, whereas, the gene expression changes induced by ethidium bromide were distinct from the other compounds (Fig 2).

The plant-derived polyphenolic compound tannic acid induced the expression of a number of putative efflux pump genes. As seen from our clustering analyses the gene expression changes induced by DIP were similar to those of tannic acid, but not as strong. DIP is a strong iron-chelator, and at least some of the antimicrobial properties of tannic acid are known to stem from its capacity for iron chelation [59]. Therefore, the overlapping expression changes induced by these compounds are most likely to be related to iron limitation in the media. A small set of genes was strongly induced by both of these compounds (Fig 2). Norfloxacin, which may also bind to metal ions [60], also caused low-level induction of the genes in this group. Most prominent among the genes induced by iron limitation was BC5182, which encodes an ABC pump similar to the *S*. *aureus* multidrug efflux pump Sav1866 (Table 3). In light of its induction by DIP and tannic acid, BC5182 may have a role in iron uptake. In line with this hypothesis a putative binding site for the ferric uptake regulator (Fur) was identified 40 nt upstream of the gene. The sequence of this Fur box (TGATAATGGTTATCA) is an almost perfect match to the Fur box sequence identified in *B*. *subtilis* [61]. The gene encoding the SecDF system, BC4405, was also weakly but specifically induced by tannic acid and DIP, which may reflect a need for the cell to re-organise its membrane protein content during iron-limitation.

Some genes, including the MFS gene BC4000 and the RND pump BC0714, appeared to be upregulated as a response to most or all of the tested conditions, although the strongest changes in expression were induced by different compounds (Fig 2). These genes may be regulated as part of general stress responses and could encode multidrug efflux pumps. The transporter encoded by BC4000 is a member of the DHA2 family of the MFS and is closely related to the characterized multidrug efflux system Bmr3 of *B*. *subtilis*, strengthening the hypothesis that this protein functions in multidrug efflux. Interestingly, the MFS efflux system encoded by BC4707, which is also closely related to Bmr3 and was recently shown to function as a multidrug exporter [23] was not highly induced by any of the compounds tested. Expression of this pump was induced by bile salts, but based on expression signals from this gene in both microarray data and qRT-PCR this gene is not constitutively expressed at a high level in *B*. *cereus* [32]. Therefore the BC4707 transport protein may have additional physiological functions that are unrelated to drug efflux.

A number of putative efflux pump genes were responsive to tetracycline and chloramphenicol exposure and fell into a single large group that may include antibiotic efflux systems (Fig 2). Many of these genes were also induced by tannic acid, albeit to a lesser extent than tetracycline (Fig 2). Notably, all three SMR family pumps, BC0358, BC0852 and BC4213 fell within this antibiotic induced group of genes and display very similar patterns of induction by the nine treatments (Fig 2). The MFS pump BC0855 was also similarly responsive to the treatments. As mentioned above genes encoding the SMR pump BC0852 and MFS pump BC0855 are preceded by a conserved palindromic sequence that could function as a binding site for a regulatory element. A similar sequence was not present in the upstream regions of the other two SMR genes or other similarly regulated genes, suggesting these genes are under the control of distinct regulatory elements. The largest transcriptional response, giving an approximately thirty-fold increase in expression compared with the untreated control, was observed for the BC0870 in response to tetracycline. BC0870 was also induced by more than ten-fold in response to chloramphenicol and by approximately three-fold in response to erythromycin. This is in line with the induction of its *B*. *subtilis* ortholog, *yheI* (*bmrC*), by ribosome targeting antibiotics (see discussion of the BC0870 promoter region above), however, kanamycin did not induce high expression.

The insect gut has been postulated to constitute a natural habitat for *B*. *cereus* group bacteria [4]. Thus, transcriptional responses for the above described transporters were analysed following exposure of *B*. *cereus* ATCC 14579 to insect antimicrobial compounds in a crude ethanol surface extract of a social paper wasp, *Polistes humilis* [24]. The putative ABC-transporter ATP-binding protein BC1359, which had only shown a minor response upon exposure to the other antimicrobial compounds tested (Fig 2), was the only pump gene showing strong expression induction by wasp extract exposure (>20-fold induction). BC1359 is encoded in a cluster of four genes that each encode an ABC transporter component (BC1357-BC1360). BC1357 and BC1359 encode nucleotide-binding domains that are most similar to ABC-2 of *Streptococcus pyogenes* and SboF of *Streptococcus salivarius*, respectively (Table 3). These nucleotide-binding domains may function with proteins encoded by BC1358 and BC1360 that each have six predicted transmembrane helices, to produce a complete transporter with 12 transmembrane helices and two nucleotide binding domains, similar to well-characterised ABC family pumps catalysing efflux. However, BC1358 and BC1360 do not display any significant similarity to characterised efflux pumps listed in the TCDB (Table 3). Additionally, the BC1358 gene is not highly conserved across the *B*. *cereus* group (20.7% conservation; Table 3), so may be dispensable or replaceable in many strains.

Based on RNA sequencing data from orthologs in *B*. *cereus* ATCC 10987, the BC1356-BC1360 cluster is likely to be co-transcribed in an operon [62]. An expanded qRT-PCR analysis of the BC1356-BC1360 locus showed that all genes were more than 19-fold upregulated following exposure to the wasp extract (S4 Table). MIC-studies further showed that Proteinase K treatment (37°C, 1 h) abolished antimicrobial activity at the maximum concentration of wasp extract available. *Polistes dominulus* has been shown to synthesize two antimicrobial peptides present on the cuticle and in the venom, Dominulin A and B, respectively [63]. A qRT-PCR experiment investigating the transcriptional response of the BC1356-BC1360 genes following exposure of *B*. *cereus* ATCC 14579 to custom synthesized Dominulin B at a concentration corresponding to 50% of its MIC value (S3 Table), showed that all genes in the locus were induced more than 26-fold (S4 Table). Interestingly this presents a novel *B*. *cereus* group transporter locus which is conserved across sequenced isolates and responds to one or more antimicrobial peptides from an insect source. This pump could constitute a case of export proteins potentially contributing to resistance to insect-derived antimicrobial peptides, a resistance type which has previously largely been attributed to alanylation of negatively charged teichoic acids by the *dlt* locus [64].

## Conclusions

Using the TransAAP we demonstrated that bacterial strains within the *B*. *cereus* group may devote more than 2.5% of their protein coding potential to the production of drug efflux pumps (Table 1). This represents one of the largest investments in efflux potential of any bacterial lineage. We have only just begun to unravel the functions associated with these many efflux systems. However, most pumps were highly conserved across the *B*. *cereus* group (Fig 1), suggesting that they mediate core functions that may be common to different species occupying a variety of niches. We suspect that a number of the efflux pumps encoded by members of the *B*. *cereus* group are able to mediate the efflux of drugs, either as a core function or fortuitously. However, due to their large numbers we have found that the characterisation of these pumps by gene deletion analyses is challenging. The work described here has highlighted putative functions for a number of pumps that warrant future focussed investigations in a heterologous system or using purified protein. For example, the BC5182 ABC pump is likely to play a role in iron homeostasis, possibly by the efflux of a siderophore, whereas BC4000 and BC0714 may represent novel multidrug efflux pumps, and the BC1357-BC1360 pump may confer resistance to antimicrobial peptides. We are particularly interested in the functional mechanisms and modes of operation of the RND superfamily pumps, such as BC0714. In Gram-negative bacteria RND efflux pumps are likely to capture their substrates from the periplasm and transport them across the outer membrane, however, their functional roles and mechanisms of transport in Gram-positive bacteria are largely unknown.

## Supporting information

S1 Fig Amino acid sequence alignment of *Bacillus* RND efflux proteins with the prototypical RND transporter AcrB from *E*. *coli*. The amino acids composing a loop likely to represent the exit site for substrates from AcrB (into TolC) is marked by a red box.
(TIFF)

S2 Fig Biofilm formation of *B*. *cereus* ATCC 14579 wild type and the isogenic Δ*secDF* deletion mutant measured in a microplate screening assay after 48h and 72h growth. (A) Bars represent the mean of four independent experiments and error bars represent the standard deviation. The *B*. *cereus* ATCC 14579 wild type is shown in dark grey and the Δ*secDF* mutant in light grey. The single star symbolizes P < 0.05 and double stars symbolize P < 0.005 in a two-tailed paired t-test. (B) Pictures show dye-stained biofilms of wild type *B*. *cereus* ATCC 14579 (B1) and Δ*secDF* (B2) strains after 48 h growth. Displayed is a top-down view of the wells, which shows a strong effect of *secDF* deletion on the submerged part of the biofilm at the bottom of the wells. Visually there was no difference in biofilm mass between the wild type and the Δ*secDF* mutant for biofilm formed in the air-liquid-interface.
(TIFF)

S1 Table *Bacillus cereus* group strains used for comparative analyses of *B*. *cereus* ATCC 14579 efflux pumps. A complete list of the 168 *B*. *cereus* group strains used in comparative analyses of efflux pumps, along with the RefSeq accession numbers of their genome sequences.
(DOCX)

S2 Table List of primers used in the current study. The names and nucleotide sequences of all primers used in the current study.
(DOCX)

S3 Table Susceptibility of *B*. *cereus* ATCC 14579 towards compounds used in antimicrobial exposure experiments. The minimum inhibitory concentrations of the compound used in transcriptional analyses against *B*. *cereus* ATCC 14579.
(DOCX)

S4 Table Expression induction of genes BC1356-BC1360 in response to wasp surface ethanol extract and Dominulin B. Relative expression of the BC1356-BC1360 gene cluster following to wasp surface ethanol extract and the antimicrobial peptide Dominulin B.
(DOCX)
