# Supplementary figures and images for: The putative drug efflux systems of the Bacillus cereus group
Source: PLoS One. 2017 May 4;12(5):e0176188. doi: 10.1371/journal.pone.0176188 (PMC5417439; doi:10.1371/journal.pone.0176188)

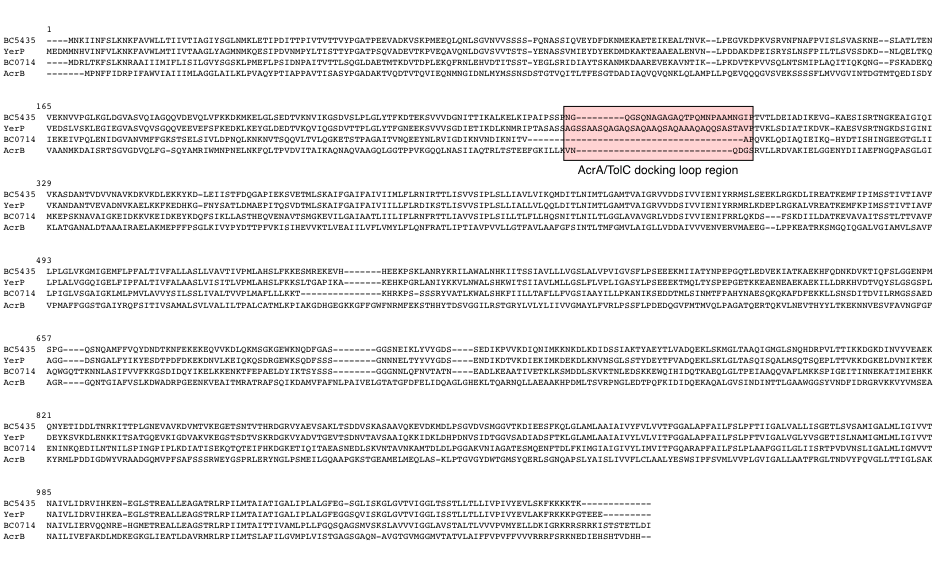

Supplement: S1 Fig — The amino acids composing a loop likely to represent the exit site for substrates from AcrB (into TolC) is marked by a red box. (TIFF) [file pone.0176188.s001.tiff]

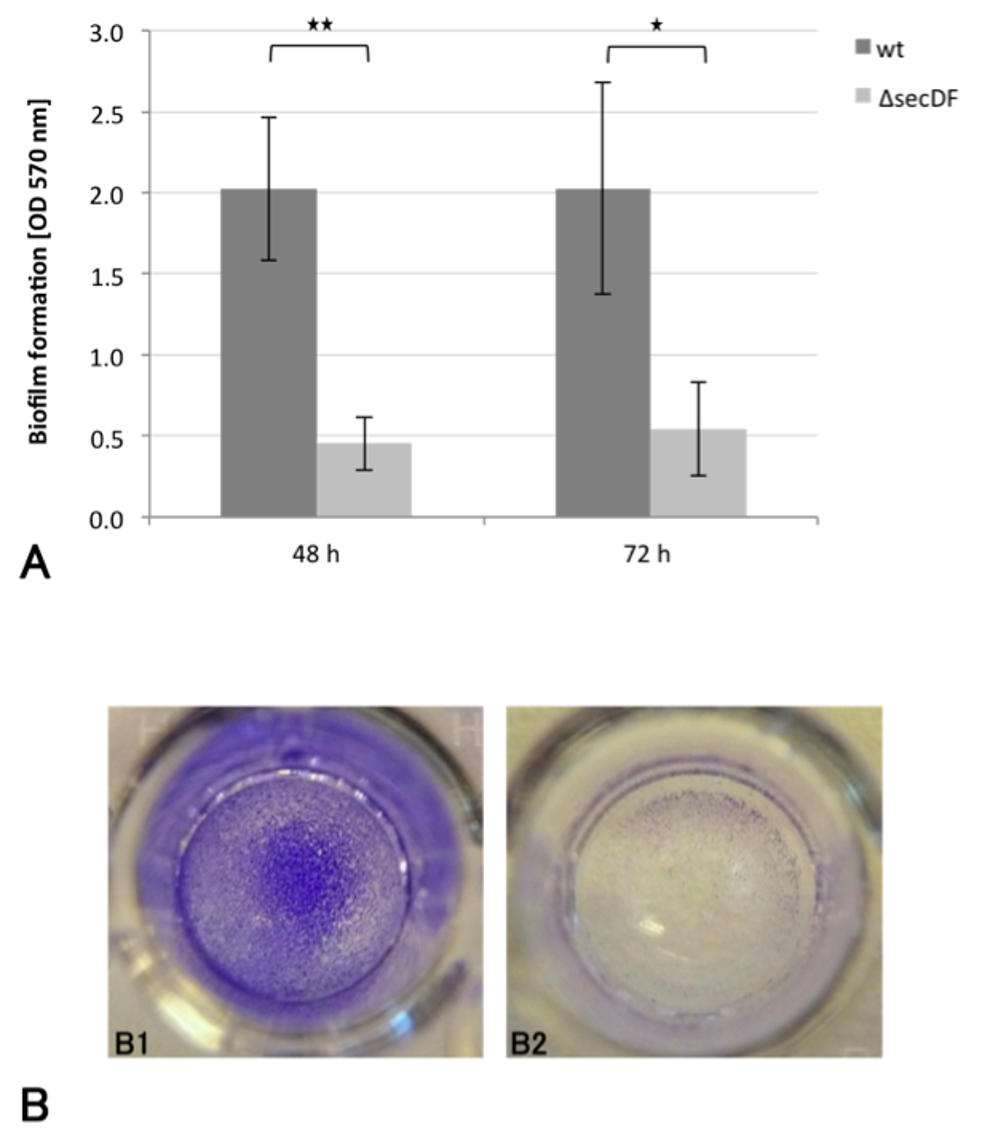

Supplement: S2 Fig — (A) Bars represent the mean of four independent experiments and error bars represent the standard deviation. The B. cereus ATCC 14579 wild type is shown in dark grey and the ΔsecDF mutant in light grey. The single star symbolizes P < 0.05 and double stars symbolize P < 0.005 in a two-tailed paired t-test. (B) Pictures show dye-stained biofilms of wild type B. cereus ATCC 14579 (B1) and ΔsecDF (B2) strains after 48 h growth. Displayed is a top-down view of the wells, which shows a strong effect of secDF deletion on the submerged part of the biofilm at the bottom of the wells. Visually there was no difference in biofilm mass between the wild type and the ΔsecDF mutant for biofilm formed in the air-liquid-interface. (TIFF) [file pone.0176188.s002.tiff]
