# Supplementary material for: The putative drug efflux systems of the Bacillus cereus group
Source: PLoS One. 2017 May 4;12(5):e0176188. doi: 10.1371/journal.pone.0176188 (PMC5417439; doi:10.1371/journal.pone.0176188)
Supplement: S1 Table — A complete list of the 168 B. cereus group strains used in comparative analyses of efflux pumps, along with the RefSeq accession numbers of their genome sequences. (DOCX) [file pone.0176188.s003.docx]

**S1 Table. *Bacillus cereus* group strains used for comparative analyses of *B. cereus* ATCC 14579 efflux pumps.**

| **Assembly acession** | **Organism name** | **Infraspecific name** | **Isolate** |
| --- | --- | --- | --- |
| GCF_000742315.1 | Bacillus anthracis | strain=Smith 1013 |  |
| GCF_000742695.1 | Bacillus anthracis | strain=delta Sterne |  |
| GCF_000742875.1 | Bacillus anthracis | strain=BFV |  |
| GCF_000808075.1 | Bacillus anthracis | strain=A0157 | NR-1041 |
| GCF_000831505.1 | Bacillus anthracis |  | Pollino |
| GCF_001277955.1 | Bacillus anthracis | strain=Larissa |  |
| GCF_000725325.1 | Bacillus anthracis | strain=HYU01 |  |
| GCF_000742655.1 | Bacillus anthracis | strain=2000031021 |  |
| GCF_000830095.1 | Bacillus anthracis | strain=Ames A0462 | NR-411 |
| GCF_000832425.1 | Bacillus anthracis | strain=PAK-1 |  |
| GCF_000832445.1 | Bacillus anthracis | strain=Vollum 1B |  |
| GCF_000832465.1 | Bacillus anthracis | strain=K3 |  |
| GCF_000832505.1 | Bacillus anthracis | strain=Ohio ACB |  |
| GCF_000832565.1 | Bacillus anthracis | strain=SK-102 |  |
| GCF_000832585.1 | Bacillus anthracis | strain=Pasteur |  |
| GCF_000832665.1 | Bacillus anthracis | strain=BA1015 |  |
| GCF_000832725.1 | Bacillus anthracis | strain=BA1035 |  |
| GCF_000832745.1 | Bacillus anthracis | strain=RA3 |  |
| GCF_000832965.1 | Bacillus anthracis | strain=2002013094 |  |
| GCF_000833065.1 | Bacillus anthracis | strain=Ames_BA1004 |  |
| GCF_000833125.1 | Bacillus anthracis | strain=Canadian Bison | A0369 |
| GCF_000875715.1 | Bacillus anthracis | strain=A1144 |  |
| GCF_001543225.1 | Bacillus anthracis | strain=Stendal |  |
| GCF_001654475.1 | Bacillus anthracis | strain=Tangail-1 |  |
| GCF_001683065.1 | Bacillus anthracis | strain=Parent2 |  |
| GCF_001683095.1 | Bacillus anthracis | strain=Parent1 |  |
| GCF_001683135.1 | Bacillus anthracis | strain=PR01 |  |
| GCF_001683155.1 | Bacillus anthracis | strain=PR02 |  |
| GCF_001683175.1 | Bacillus anthracis | strain=PR05 |  |
| GCF_001683195.1 | Bacillus anthracis | strain=PR06 |  |
| GCF_001683215.1 | Bacillus anthracis | strain=PR07 |  |
| GCF_001683235.1 | Bacillus anthracis | strain=PR08 |  |
| GCF_001683255.1 | Bacillus anthracis | strain=PR09-1 |  |
| GCF_001683275.1 | Bacillus anthracis | strain=PR09-4 |  |
| GCF_001683295.1 | Bacillus anthracis | strain=PR10-4 |  |
| GCF_000559005.1 | Bacillus anthracis 52-G | strain=52-G |  |
| GCF_000558965.1 | Bacillus anthracis 8903-G | strain=8903-G |  |
| GCF_000558985.1 | Bacillus anthracis 9080-G | strain=9080-G |  |
| GCF_000008445.1 | Bacillus anthracis str. 'Ames Ancestor' | strain=Ames Ancestor |  |
| GCF_000022865.1 | Bacillus anthracis str. A0248 | strain=A0248 |  |
| GCF_000512835.1 | Bacillus anthracis str. A16 | strain=A16 |  |
| GCF_000512775.1 | Bacillus anthracis str. A16R | strain=A16R |  |
| GCF_000007845.1 | Bacillus anthracis str. Ames | strain=Ames |  |
| GCF_000021445.1 | Bacillus anthracis str. CDC 684 | strain=CDC 684 |  |
| GCF_000258885.1 | Bacillus anthracis str. H9401 | strain=H9401 |  |
| GCF_000008165.1 | Bacillus anthracis str. Sterne | strain=Sterne |  |
| GCF_000832635.1 | Bacillus anthracis str. Sterne | strain=Sterne |  |
| GCF_000583105.1 | Bacillus anthracis str. SVA11 | strain=SVA11 |  |
| GCF_000833275.1 | Bacillus anthracis str. Turkey32 | strain=Turkey32 |  |
| GCF_000832785.1 | Bacillus anthracis str. V770-NP-1R | strain=V770-NP-1R |  |
| GCF_000742895.1 | Bacillus anthracis str. Vollum | strain=Vollum |  |
| GCF_000635895.2 | Bacillus cereus | strain=A1 |  |
| GCF_000789315.1 | Bacillus cereus | strain=03BB87 |  |
| GCF_000832525.1 | Bacillus cereus | strain=FM1 |  |
| GCF_000832765.1 | Bacillus cereus | strain=3a |  |
| GCF_000835185.1 | Bacillus cereus | strain=S2-8 |  |
| GCF_000978375.1 | Bacillus cereus | strain=FORC_005 | chicken cutlett |
| GCF_001277915.1 | Bacillus cereus | strain=NJ-W |  |
| GCF_001518875.1 | Bacillus cereus | strain=FORC_013 |  |
| GCF_001635915.1 | Bacillus cereus | strain=CMCC P0021 |  |
| GCF_001635955.1 | Bacillus cereus | strain=CMCC P0011 |  |
| GCF_001635995.1 | Bacillus cereus | strain=HN001 |  |
| GCF_000022505.1 | Bacillus cereus 03BB102 | strain=03BB102 |  |
| GCF_000832405.1 | Bacillus cereus 03BB102 | strain=03BB102 |  |
| GCF_000832865.1 | Bacillus cereus 03BB108 | strain=03BB108 |  |
| GCF_000160935.1 | Bacillus cereus 172560W | strain=172560W |  |
| GCF_000161135.1 | Bacillus cereus 95/8201 | strain=95/8201 |  |
| GCF_000161375.1 | Bacillus cereus AH1271 | strain=AH1271 |  |
| GCF_000161395.1 | Bacillus cereus AH1272 | strain=AH1272 |  |
| GCF_000003955.1 | Bacillus cereus AH1273 | strain=AH1273 |  |
| GCF_000021225.1 | Bacillus cereus AH187 | strain=AH187 |  |
| GCF_000161335.1 | Bacillus cereus AH603 | strain=AH603 |  |
| GCF_000160975.1 | Bacillus cereus AH621 | strain=AH621 |  |
| GCF_000161355.1 | Bacillus cereus AH676 | strain=AH676 |  |
| GCF_000021785.1 | Bacillus cereus AH820 | strain=AH820 |  |
| GCF_000160895.1 | Bacillus cereus ATCC 10876 | strain=ATCC 10876 |  |
| GCF_000008005.1 | Bacillus cereus ATCC 10987 | strain=ATCC 10987 |  |
| *GCF_000007825.1 | Bacillus cereus ATCC 14579 | strain=ATCC 14579 |  |
| GCF_000161015.1 | Bacillus cereus ATCC 4342 | strain=ATCC 4342 |  |
| GCF_000832845.1 | Bacillus cereus ATCC 4342 | strain=ATCC 4342 |  |
| GCF_000021205.1 | Bacillus cereus B4264 | strain=B4264 |  |
| GCF_000161115.1 | Bacillus cereus BDRD-Cer4 | strain=BDRD-Cer4 |  |
| GCF_000161095.1 | Bacillus cereus BDRD-ST196 | strain=BDRD-ST196 |  |
| GCF_000161055.1 | Bacillus cereus BDRD-ST24 | strain=BDRD-ST24 |  |
| GCF_000161075.1 | Bacillus cereus BDRD-ST26 | strain=BDRD-ST26 |  |
| GCF_000160915.1 | Bacillus cereus BGSC 6E1 | strain=BGSC 6E1 |  |
| GCF_000143605.1 | Bacillus cereus biovar anthracis str. CI | strain=CI |  |
| GCF_000832385.1 | Bacillus cereus D17 | strain=D17 |  |
| GCF_000011625.1 | Bacillus cereus E33L | strain=E33L |  |
| GCF_000833045.1 | Bacillus cereus E33L | strain=E33L |  |
| GCF_000338315.1 | Bacillus cereus F | strain=F |  |
| GCF_000161315.1 | Bacillus cereus F65185 | strain=F65185 |  |
| GCF_000239195.1 | Bacillus cereus F837/76 | strain=F837/76 |  |
| GCF_000292415.1 | Bacillus cereus FRI-35 | strain=FRI-35 |  |
| GCF_000832805.1 | Bacillus cereus G9241 | strain=G9241 |  |
| GCF_000021305.1 | Bacillus cereus G9842 | strain=G9842 |  |
| GCF_000003645.1 | Bacillus cereus m1293 | strain=m1293 |  |
| GCF_000161035.1 | Bacillus cereus m1550 | strain=m1550 |  |
| GCF_000160955.1 | Bacillus cereus MM3 | strain=MM3 |  |
| GCF_000283675.1 | Bacillus cereus NC7401 |  |  |
| GCF_000013065.1 | Bacillus cereus Q1 | strain=Q1 |  |
| GCF_000160995.1 | Bacillus cereus R309803 | strain=R309803 |  |
| GCF_000161175.1 | Bacillus cereus Rock1-15 | strain=Rock1-15 |  |
| GCF_000161155.1 | Bacillus cereus Rock1-3 | strain=Rock1-3 |  |
| GCF_000161195.1 | Bacillus cereus Rock3-28 | strain=Rock3-28 |  |
| GCF_000161215.1 | Bacillus cereus Rock3-29 | strain=Rock3-29 |  |
| GCF_000161235.1 | Bacillus cereus Rock3-42 | strain=Rock3-42 |  |
| GCF_000161255.1 | Bacillus cereus Rock3-44 | strain=Rock3-44 |  |
| GCF_000161295.1 | Bacillus cereus Rock4-18 | strain=Rock4-18 |  |
| GCF_000161275.1 | Bacillus cereus Rock4-2 | strain=Rock4-2 |  |
| GCF_000017425.1 | Bacillus cytotoxicus NVH 391-98 | strain=NVH 391-98 |  |
| GCF_000742855.1 | Bacillus mycoides | strain=219298 |  |
| GCF_000832605.1 | Bacillus mycoides | strain=ATCC 6462 |  |
| GCF_000003925.1 | Bacillus mycoides DSM 2048 | strain=DSM 2048 |  |
| GCF_000161415.1 | Bacillus mycoides Rock1-4 | strain=Rock1-4 |  |
| GCF_000161435.1 | Bacillus mycoides Rock3-17 | strain=Rock3-17 |  |
| GCF_000161455.1 | Bacillus pseudomycoides DSM 12442 | strain=DSM 12442 |  |
| GCF_000832485.1 | Bacillus thuringiensis | strain=HD1011 |  |
| GCF_000832825.1 | Bacillus thuringiensis | strain=HD571 |  |
| GCF_000832925.1 | Bacillus thuringiensis | strain=HD682 |  |
| GCF_000833085.1 | Bacillus thuringiensis | strain=97-27 |  |
| GCF_001017635.1 | Bacillus thuringiensis | strain=YC-10 |  |
| GCF_001182785.1 | Bacillus thuringiensis | strain=HS18-1 |  |
| GCF_001420855.1 | Bacillus thuringiensis | strain=YWC2-8 |  |
| GCF_001455345.1 | Bacillus thuringiensis | strain=CTC |  |
| GCF_001595725.1 | Bacillus thuringiensis | strain=Bt185 |  |
| GCF_001598095.1 | Bacillus thuringiensis | strain=HD12 |  |
| GCF_001618665.1 | Bacillus thuringiensis | strain=Bc601 |  |
| GCF_001685565.1 | Bacillus thuringiensis | strain=MYBT18246 |  |
| GCF_001692675.1 | Bacillus thuringiensis | strain=KNU-07 |  |
| GCF_000092165.1 | Bacillus thuringiensis BMB171 | strain=BMB171 |  |
| GCF_000161495.1 | Bacillus thuringiensis Bt407 | strain=Bt407 |  |
| GCF_000306745.1 | Bacillus thuringiensis Bt407 |  |  |
| GCF_000342025.1 | Bacillus thuringiensis DAR 81934 | strain=DAR 81934 |  |
| GCF_000292455.1 | Bacillus thuringiensis HD-771 | strain=HD-771 |  |
| GCF_000292705.1 | Bacillus thuringiensis HD-789 | strain=HD-789 |  |
| GCF_000835025.1 | Bacillus thuringiensis HD1002 | strain=HD1002 |  |
| GCF_000161715.1 | Bacillus thuringiensis IBL 200 | strain=IBL 200 |  |
| GCF_000161735.1 | Bacillus thuringiensis IBL 4222 | strain=IBL 4222 |  |
| GCF_000300475.1 | Bacillus thuringiensis MC28 | strain=MC28 |  |
| GCF_001640965.1 | Bacillus thuringiensis serovar alesti | strain=BGSC 4C1 |  |
| GCF_000161635.1 | Bacillus thuringiensis serovar andalousiensis BGSC 4AW1 | strain=BGSC 4AW1 |  |
| GCF_000161615.1 | Bacillus thuringiensis serovar berliner ATCC 10792 | strain=ATCC 10792 |  |
| GCF_000193355.1 | Bacillus thuringiensis serovar chinensis CT-43 |  |  |
| GCF_000190515.1 | Bacillus thuringiensis serovar finitimus YBT-020 |  |  |
| GCF_000803665.1 | Bacillus thuringiensis serovar galleriae | strain=4G5 |  |
| GCF_000161675.1 | Bacillus thuringiensis serovar huazhongensis BGSC 4BD1 | strain=BGSC 4BD1 |  |
| GCF_001183785.1 | Bacillus thuringiensis serovar indiana | strain=HD521 |  |
| GCF_000008505.1 | Bacillus thuringiensis serovar konkukian str. 97-27 | strain=97-27 |  |
| GCF_000835235.1 | Bacillus thuringiensis serovar kurstaki | strain=HD 1i |  |
| GCF_000717535.1 | Bacillus thuringiensis serovar kurstaki str. HD-1 | strain=HD-1 |  |
| GCF_000338755.1 | Bacillus thuringiensis serovar kurstaki str. HD73 | strain=HD73 |  |
| GCF_000161575.1 | Bacillus thuringiensis serovar kurstaki str. T03a001 | strain=T03a001 |  |
| GCF_000688795.1 | Bacillus thuringiensis serovar kurstaki str. YBT-1520 | strain=YBT-1520 |  |
| GCF_000747545.1 | Bacillus thuringiensis serovar kurstaki str. YBT-1520 | strain=YBT-1520 |  |
| GCF_000161595.1 | Bacillus thuringiensis serovar monterrey BGSC 4AJ1 | strain=BGSC 4AJ1 |  |
| GCF_000940785.1 | Bacillus thuringiensis serovar morrisoni | strain=serovar morrisoni BGSC 4AA1 |  |
| GCF_000161555.1 | Bacillus thuringiensis serovar pakistani str. T13001 | strain=T13001 |  |
| GCF_000161655.1 | Bacillus thuringiensis serovar pondicheriensis BGSC 4BA1 | strain=BGSC 4BA1 |  |
| GCF_000161695.1 | Bacillus thuringiensis serovar pulsiensis BGSC 4CC1 | strain=BGSC 4CC1 |  |
| GCF_000341665.1 | Bacillus thuringiensis serovar thuringiensis str. IS5056 | strain=IS5056 |  |
| GCF_000161515.1 | Bacillus thuringiensis serovar thuringiensis str. T01001 | strain=T01001 |  |
| GCF_000161475.1 | Bacillus thuringiensis serovar tochigiensis BGSC 4Y1 | strain=BGSC 4Y1 |  |
| GCF_001548175.1 | Bacillus thuringiensis serovar tolworthi |  |  |
| GCF_000015065.1 | Bacillus thuringiensis str. Al Hakam | strain=Al Hakam |  |
| GCF_000832885.1 | Bacillus thuringiensis str. Al Hakam | strain=Al Hakam |  |
| GCF_000497525.1 | Bacillus thuringiensis YBT-1518 | strain=YBT-1518 |  |
| GCF_000775975.1 | Bacillus weihenstephanensis | strain=WSBC10204 |  |
| GCF_000018825.1 | Bacillus weihenstephanensis KBAB4 | strain=KBAB4 |  |

* *B. cereus* ATCC 14579 was used as the reference isolate.

The table incudes relevant information from the NCBI RefSeq assembly summary table.
