# Supplementary material for: The putative drug efflux systems of the Bacillus cereus group
Source: PLoS One. 2017 May 4;12(5):e0176188. doi: 10.1371/journal.pone.0176188 (PMC5417439; doi:10.1371/journal.pone.0176188)
Supplement: S2 Table — The names and nucleotide sequences of all primers used in the current study. (DOCX) [file pone.0176188.s004.docx]

**S2 Table. List of primers used in the current study**

| Forward primer | Sequence 5' to 3' | Reverse primer | Sequence 5' to 3' |
| --- | --- | --- | --- |
| BC0256_MFS_F | cagctgcatcagctatggtc | BC0256_MFS_R | ccaatcgcagcacctatatt |
| BC0434_MFS_F | tatgctcgttatggcgtctg | BC0434_MFS_R | accggatcattattcgttcg |
| BC0667_MFS_F | tatctggtgctgctgttgga | BC0667_MFS_R | cgatcgtaccaagagccatt |
| BC0855_MFS_F | ggattaatcattccggttatgc | BC0855_MFS_R | ccatcggcctgtaataggtg |
| BC1681_MFS_F | gcattaacttcgtctattccgagt | BC1681_MFS_R | aaccatatacaactccgccaat |
| BC1786_MFS_F | tatcgccaatatggggaaag | BC1786_MFS_R | acaaaccccataagcgtcat |
| BC2230_MFS_F | aagagagtggaggagagacaaca | BC2230_MFS_R | ccgattccttcgttacatagc |
| BC2894_MFS_F | cttggcacaggcttcttctt | BC2894_MFS_R | gcagattgcgaatgctgtt |
| BC3212_MFS_F | tggtgatgatgccacttatga | BC3212_MFS_R | gtaccaatggaaccggacac |
| BC3310_MFS_F | aaacatggatcacgacgaca | BC3310_MFS_R | accgtactgcacagtgcttg |
| BC4000_MFS_F | tttgtttgggtcacatcagc | BC4000_MFS_R | gaaaagaatgaggccaccaa |
| BC4568_MFS_F | gatgatgacaggtcgcgtaa | BC4568_MFS_R | cgtttatgaggcgggaataa |
| BC4707_MFS_F | acggaaagctcgctgattta | BC4707_MFS_R | gcgcggaagaagattaattg |
| BC0852_SMR_F | cggagctggtacggtaggta | BC0852_SMR_R | aaccgataacgccagctaca |
| BC0358_SMR_F | aagctcgttccaagtgtactga | BC0358_SMR_R | taatgttccaacgccagacc |
| BC4213_SMR_F | agcagaggcaccacttgaat | BC4213_SMR_R | accagctccgattcctgtaa |
| BC1383_MATE_F | ctattgcagctcaccaagca | BC1383_MATE_R | gctccaacttcgaatccaac |
| BC1615_MATE_F | aataccagccgttcttggaa | BC1615_MATE_R | atgctggaatagccacgttc |
| BC1716_MATE_F | gctcggtattccagcgagta | BC1716_MATE_R | cttgattcacaacgccgtaa |
| BC1184_MATE_F | gctcgcaatgaacttacaagg | BC1184_MATE_R | acggataattgcggctaatg |
| BC0714_RND_F | accgagctgccattatcatc | BC0714_RND_R | tgtgacggtaattgctggatt |
| BC1291_RND_F | agatgcttcgcatgaggaat | BC1291_RND_R | gccgatgtcatcttgtagca |
| BC4405_RND_F | cgtgtacagcttgctggtgt | BC4405_RND_R | ccgttccatccataagaagg |
| BC5435_RND_F | caatgattggtgcgcttatg | BC5435_RND_R | cgtgttgcaccagcttctaa |
| BC1356_F | ccctctgtccgtgaattagc | BC1356_R | taccgtcccattcctctacg |
| BC1357_ABC_F | tgtccgataagccaatctttg | BC1357_ABC_R | aaattctgcaatcgtatctaccg |
| BC1358_ABC_F | caggagttagaatttcaggtaggc | BC1358_ABC_R | tttccgaaaagacgatatacctg |
| BC1359_ABC_F | aagagcgcattgctgagatt | BC1359_ABC_R | atgcagtaatgcctgtgcaa |
| BC1360_ABC_F | gttgaagtggggaaaaggtg | BC1360_ABC_R | aagagtacgggcagcaagtg |
| BC5285_ABC_F | ggaccgagtggatctggtaa | BC5285_ABC_R | tcgatgctacttggtcctgt |
| BC0509_ABC_F | caaggaatgcaagtgacacg | BC0509_ABC_R | aatgttcttgccgtccaact |
| BC0870_ABC_F | gtacggaagcagcgatcatt | BC0870_ABC_R | actgcgccttcatccataac |
| BC5182_ABC_F | gctcacagacttgcaacgat | BC5182_ABC_R | tactgtatcctccgccttgc |
| BC5433_ABC_F | ttagcaggtgaacacgttgg | BC5433_ABC_R | gtgtccattctacgcgacct |
| helicase_F | cgagaaaagaaactgcccata | helicase_R | gctctgcttgaattccatctg |
