# Supplementary material for: The putative drug efflux systems of the Bacillus cereus group
Source: PLoS One. 2017 May 4;12(5):e0176188. doi: 10.1371/journal.pone.0176188 (PMC5417439; doi:10.1371/journal.pone.0176188)
Supplement: S3 Table — The minimum inhibitory concentrations of the compound used in transcriptional analyses against B. cereus ATCC 14579. (DOCX) [file pone.0176188.s005.docx]

**S3 Table. Susceptibility of *B. cereus* ATCC 14579 towards compounds used in antimicrobial exposure experiments**

| Substance | Class/comment | MIC |
| --- | --- | --- |
| Chloramphenicol | Phenicol antibiotic | 2.5 μg/ml* |
| Norfloxacin | Quinolone antibiotic | 2.5 µg/ml |
| Kanamycin | Aminoglycoside antibiotic | 15 μg/ml* |
| Erythromycin | Macrolide antibiotic | 0.2 μg/ml* |
| Tetracycline | Tetracycline antibiotic | 2.5 μg/ml* |
| Ethidium bromide | Antimicrobial dye, common MD efflux pump substrate | 40 μg/ml* |
| 2,2’-dipyridol | Iron chelator | 1 mM |
| Tannic acid | Plant derived polyphenol, iron chelator | 40 µg/ml |
| Dominulin B | Insect derived antimicrobial peptide | 16 µg/ml |

* The MIC was previously determined [23].
