# Supplementary material for: The putative drug efflux systems of the Bacillus cereus group
Source: PLoS One. 2017 May 4;12(5):e0176188. doi: 10.1371/journal.pone.0176188 (PMC5417439; doi:10.1371/journal.pone.0176188)
Supplement: S4 Table — Relative expression of the BC1356-BC1360 gene cluster following to wasp surface ethanol extract and the antimicrobial peptide Dominulin B. (DOCX) [file pone.0176188.s006.docx]

**S4 Table. Expression induction of genes BC1356-BC1360 in response to wasp surface ethanol extract and Dominulin B.**

| Gene | Relative expression following wasp surface ethanol extract exposure | Relative expression following exposure of cells to Dominulin B |
| --- | --- | --- |
| BC1356 | 19.4 (+/- 2.2) | 81.4 (+/- 2.4) |
| BC1357 | 22.4 (+/- 1.7) | 67.8 (+/- 2.2) |
| BC1358 | 27.9 (+/- 1.3) | 48.4 (+/- 1.6) |
| BC1359 | 23.2 (+/- 1.7) | 26.1 (+/- 2.2) |
| BC1360 | 20.4 (+/- 1.5) | 26.8 (+/- 1.9) |

* Expression was normalised to that of the BC1744 helicase gene, and the relative expression shown is the geometric mean of three independent experiments. The standard deviation indicated in parentheses is the geometric standard deviation.
